# The effect of long-range interactions on the infrared and Raman spectra of aragonite (CaCO_3_, *Pmcn*) up to 25 GPa

**DOI:** 10.1038/s41598-023-29783-7

**Published:** 2023-02-15

**Authors:** Gianfranco Ulian, Giovanni Valdrè

**Affiliations:** Dipartimento di Scienze Biologiche, Geologiche e Ambientali, Centro di Ricerche Interdisciplinari di Biomineralogia, Cristallografia e Biomateriali, Università di Bologna “Alma Mater Studiorum” Piazza di Porta, San Donato 1, 40126 Bologna, Italy

**Keywords:** Density functional theory, Infrared spectroscopy, Raman spectroscopy, Mineralogy

## Abstract

Long-range interactions are relevant in the physical description of materials, even for those where other stronger bonds give the leading contributions. In this work, we demonstrate this assertion by simulating the infrared and Raman spectra of aragonite, an important calcium carbonate polymorph (space group *Pmcn*) in geological, biological and materials science fields. To this aim, we used Density Functional Theory methods and two corrections to include long-range interactions (DFT-D2 and DFT-D3). The results were correlated to IR spectroscopy and confocal Raman spectrometry data, finding a very good agreement between theory and experiments. Furthermore, the evolution of the IR/Raman modes up to 25 GPa was described in terms of mode-Grüneisen’s parameters, which are useful for geological and materials science applications of aragonite. Our findings clearly show that weak interactions are of utmost importance when modelling minerals and materials, even when they are not the predominant forces.

## Introduction

Despite the robustness and wide acceptance of Kohn–Sham density functional theory (DFT) in predicting the properties of zero- to three-dimensional systems, almost three decades ago it was demonstrated that all common functionals, including hybrids, are not able to properly describe long-range interactions, such as van der Waals (vdW) forces^1^. These dispersive interactions due to pure electronic correlation effects (London dispersion energy) at large interatomic distances are fundamental for several physical and chemical properties of matter and contribute to the description of, to cite some examples, the structures of both organic (e.g., DNA and proteins) and inorganic (e.g., crystals) substances, and the adsorption of molecules onto surfaces. Interestingly, it was shown that standard DFT approaches “under-correlate” even at short distances, i.e., some energy contribution to the total energy is missing also at distances typical of chemical bonds (covalent, ionic)^2–6^. Thus, the proper treatment of correlation effects via ad hoc corrections to the local DFT functionals is non-trivial even for solid phases where dispersive interactions do not play a leading role.

**Figure 1:**
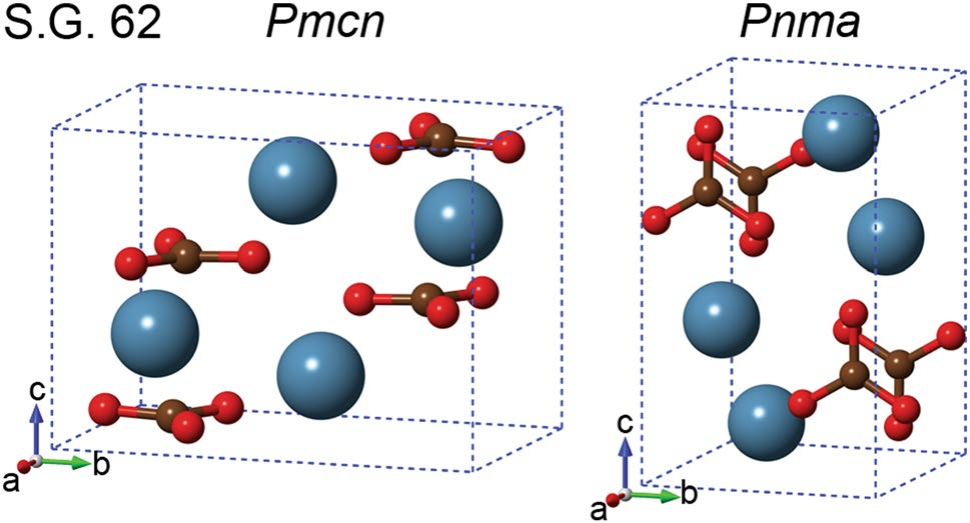
Aragonite unit cell, according to the *Pmcn* (on the left, the one employed in the present work) and to the *Pnma* (on the right) crystallographic settings. The reference frame was oriented so that the *x*, *y* and *z* Cartesian axes are parallel to the *a*, *b* and *c* lattice vectors, respectively. Calcium, carbon and oxygen atoms in the unit cell are shown as blue, ochre and red spheres, respectively.

Recently, we showed that this consideration is true for the low-pressure polymorph of CaCO_3_, *i.e.* calcite (trigonal lattice, space group $R\overline{3}c$, no. 167)^7–9^, and here we extend this approach to aragonite (orthorhombic, s.g. *Pmcn*, no. 62, Fig. 1). According to a recent experimental and theoretical thermodynamics study^10^, calcite is the stable phase at room conditions (1 atm, 300 K), whereas aragonite is a high-pressure phase (calcite-aragonite phase transition at 300 K of about 0.3 GPa). From the geological perspective, carbonates are considered possible carbon carriers for the creation of deep carbon reservoirs in the interior of Earth^11,12^. While calcite is the widest calcium carbonate, formed in sedimentary, igneous and metamorphic rocks, aragonite is mostly the product of biomineralization processes^13^. For example, the polyps of corals grow and organize aragonite crystals in marine (liquid) environments, forming intricate skeletal structures^14^, a biologically-driven process that is aided by organic molecules known as templates^15^. In general, aragonite is by far the most common phase occurring as a biomineral in many invertebrate skeletons and sediments derived from them. Furthermore, aragonite is found in the deposition of hot springs, and in stalactite/stalagmite cave formations^13^. At the industrial level, calcium carbonates are also important additives used in medicines, building materials, polymers, and many other applications^16^.

For all these reasons, aragonite and the other CaCO_3_ phases were thoroughly investigated with different techniques, such as X-ray diffraction (XRD)^17–19^, scanning electron microscopy^20,21^, cathodoluminescence^22–24^, infrared (IR) and Raman spectroscopies^25,26^ and also ab initio methods, including Density Functional Theory^25–27^. However, most of the cited theoretical works were conducted with local functionals that do not consider the effects of long-range interactions. An interesting result of previous DFT simulations on aragonite is that the unit cell volume was overestimated with respect to the X-ray diffraction refinements reported in the literature^19^. The same overestimation was observed by both generalized-gradient approximation functionals and hybrid ones for calcite^7^. This is a non-expected behaviour because static DFT simulations are carried out at absolute zero Kelvin, namely no thermal effects are included in the computations, hence the theoretical unit cell volume should be smaller than the experimental ones. This is also reflected in the computed vibrational frequencies, most of them being underestimated, i.e., red-shifted in comparison with the experimental IR and Raman data, for both low-pressure and high-pressure calcium carbonate polymorphs.

As previously introduced, we aim at demonstrating that long-range interactions are important for aragonite, considering a combined theoretical and experimental analysis of the infrared and Raman spectra of this high-pressure phase. We adopted DFT for the computational part, using the well-known B3LYP hybrid functional and the two DFT-D*n* corrections (*n* = 2, 3) to add the contributions of van der Waals interactions. Since the dispersion parameters of the B3LYP-D2 approach were recently optimized in the so-called B3LYP-D* method, this latter notation will be employed to refer to the specific DFT-D2 correction. The two methods are formally similar, namely they add an a posteriori contribution (*E*_vdW_) to the total energy according to the formula *E*_tot_ = *E*_DFT_ + *E*_vdW_. The main difference between the two methods resides in the atomic parameters used in the formulations, which are fixed in DFT-D2 and dependent on the geometry of the system in DFT-D3. This means that B3LYP-D3 is slightly less empirical than the B3LYP-D*. These two approaches were employed in a previous work of us^28^, where we obtained a better description of the unit cell of aragonite by including these missing interactions in the physical treatment via the a posteriori DFT-D*n* corrections, with the B3LYP-D3 approach resulting in a cell volume smaller than that resulting from XRD refinements. This is due to a significant shortening of the Ca–O distances and, to lesser extent, of the C–O bond lengths, meaning that the interaction between Ca^2+^ and CO_3_^2−^ ions increased. Some brief structural results are reported in Table S1 in the Supplementary Materials for the sake of completeness. Since the total energy of the system changes due to the inclusion of the London interactions, also the vibrational frequencies are expected to show variations because they are calculated from the Hessian matrix, *i.e.*, the second derivatives of the energy with respect to the atomic displacements. The IR/Raman results obtained in the present work were assessed using experimental data reported in the literature, and also polycrystalline Raman spectra of powdered aragonite acquired in the context of this study. Also, we investigated the evolution of the vibrational (optical) modes of the mineral with pressure between − 3 GPa and 25 GPa. Up to now, there is only a single theoretical work on this last topic, which reported a striking variation of two modes with the cell volume, suggesting a possible phonon instability at certain *PT* conditions^27^. As it will be shown below, we checked if this behaviour is due to the absence of proper treatment of long-range interactions, or if it is subtly related to specific computational parameters of the simulations.

## Results and discussion

### Infrared and Raman spectra at 0 GPa

In this section, a comparison between the infrared and Raman vibrational modes and spectra of aragonite simulated in this work at equilibrium and the data reported in theoretical/experimental literature is performed to assess the quality of the DFT approach adopted in the present work. It is worth mentioning that the vibrational frequencies were obtained within the harmonic approximation, whereas experimental ones are intrinsically anharmonic. Hence, all DFT results will be systematically blue-shifted, i.e., they will show higher frequency wavenumbers than experimental data.

The aragonite crystallographic cell contains four unit formula (*Z* = 4) of CaCO_3_, for a total of *N* = 20 atoms and 3*N* = 60 degrees of freedom. Since the mineral belongs to the space group *Pmcn* (point group *D*_2h_, crystal class *mmm*), three atoms (Ca, C, O1) occupy a 4*c* site and the O2 atom resides in the Wyckoff position 8*d*, the total irreducible representation (IRREP) of the degrees of freedom (*Γ*_tot_) is given by:

$$\Gamma_{{{\text{tot}}}} \, = \,\Gamma_{{\text{a}}} \, + \,\Gamma_{{\text{o}}} \, = \,{9}A_{{\text{g}}} \, + \,{6}A_{{\text{u}}} \, + \,{6}B_{{{\text{1g}}}} \, + \,{9}B_{{{\text{1u}}}} \, + \,{9}B_{{{\text{2g}}}} \, + \,{6}B_{{{\text{2u}}}} \, + \,{6}B_{{{\text{3g}}}} \, + \,{9}B_{{{\text{3u}}}} ,$$

that can be subdivided in acoustic (*Γ*_a_ = *B*_1u_ + *B*_2u_ + *B*_3u_), infrared-active (8*B*_1u_ + 5*B*_2u_ + 8*B*_3u_), Raman-active (9*A*_g_ + 6*B*_1g_ + 9*B*_2g_ + 6*B*_3g_) and silent (6*A*_u_). The subscripts *g* and *u* indicate *gerade* and *ungerade* modes that are, respectively, symmetric or antisymmetric with respect to the inversion centre. In addition, the presence of this symmetry element activates the so-called mutual exclusion rule, hence vibrational modes are active only in infrared (IRREPs *B*_1u_, *B*_2u_ and *B*_3u_) or in Raman (*A*_g_, *B*_1g_, *B*_2g_ and *B*_3g_), but not in both.

The optical modes are further distinguished in 33 “external”, meaning rotation and translation of the carbonate group and calcium ion (labelled as lattice modes, “L”), and 24 “internal”, namely the vibrational modes of the CO_3_^2−^ ions. According to the assignment proposed by White^29^, and from a graphical inspection of the internal optical modes, the carbonate group vibrations are subdivided into in-plane (labelled as ν_4_, between 700 cm^−1^ and 720 cm^−1^) and out-of-plane (ν_2_, 850–920 cm^−1^) O–C–O bending modes, and symmetric (ν_1_, between 1080 cm^−1^ and 1090 cm^−1^) and asymmetric (ν_3_, about 1440–1600 cm^−1^) C–O stretching motions.

**Figure 2:**
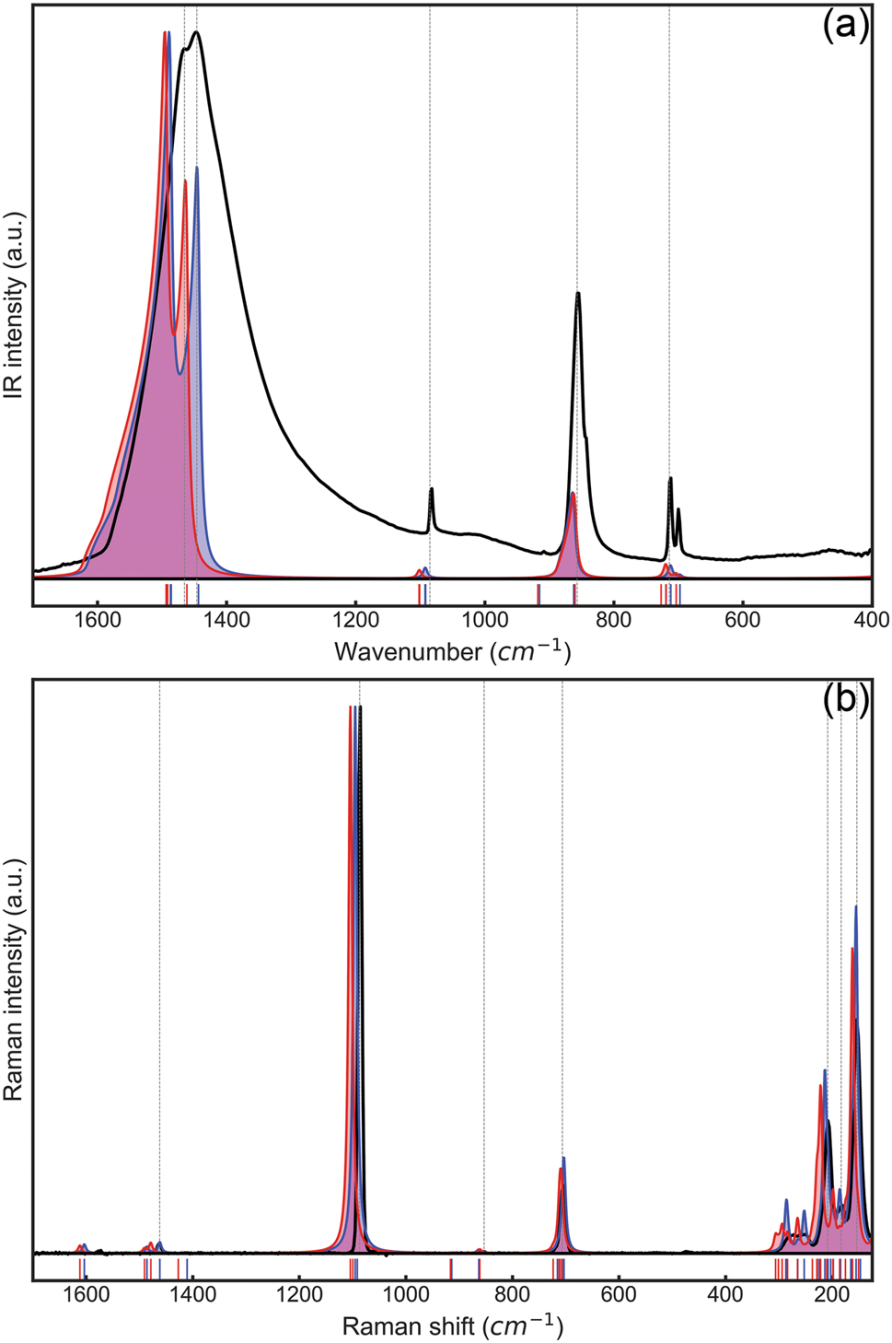
(**a**) Infrared and (**b**) Raman spectra of aragonite, as simulated at the B3LYP-D* (filled blue line) and B3LYP-D3 (filled red line) levels of theory. Experimental IR^25^ and Raman (present work) spectra are reported as black lines for a direct comparison. The small blue and red vertical lines mark the simulated vibrational frequencies with the B3LYP-D* and B3LYP-D3 approaches, respectively, whereas the grey dashed ones highlight the experimental ones. Intensities were normalized to the most intense peak in the spectra.

The B3LYP-D* and B3LYP-D3 results of the infrared vibrational frequencies are reported in Table 1, alongside previous simulations and experimental measurements, whereas Fig. 2a shows the calculated IR spectrum of aragonite. As expected, all the modes are affected by the DFT-D*n* corrections, because the total energy calculated for each atomic displacement include *E*_vdW_, hence they contribute to the construction of the Hessian matrix. The extent of their contribution is different according to which atoms are involved in the specific vibrational mode, with those at low wavenumbers (involving mostly Ca^2+^ ions and CO_3_^2–^ rotations) being the more affected (up to about 50 cm^−1^). Only slight variations were observed for carbonate ions bending and symmetric stretching modes (less than about 10 cm^−1^ for B3LYP-D3), but the higher variations were noted for the asymmetric stretching modes. Furthermore, considering the B3LYP data as the reference^25,26^, the B3LYP-D3 frequencies are more blue-shifted than the B3LYP-D* ones. The vibrational results follow what was observed on the equilibrium geometry of aragonite in our previous work^28^ and, thus, should be more in line with the physics of the system at absolute zero. The optical frequencies are in general good agreement with those of the detailed experimental/theoretical work of Carteret and collaborators^25^, showing relatively low absolute variations. More into details, the mean absolute deviations (MADs) of the modes with IRREP *B*_1u_, *B*_2u_ and *B*_3u_ simulated with the B3LYP-D* (B3LYP-D3) approach are 5.8 cm^−1^ (16.3 cm^−1^), 20.0 cm^−1^ (11.5 cm^−1^) and 7.8 cm^−1^ (16.0 cm^−1^), respectively, in line with the previous B3LYP simulations performed without the inclusion of dispersive interactions^25^. It is interesting noting that the DFT-D2 scheme provided a mix of blue- and red-shifted IR peaks, *i.e.*, the calculated modes were at higher and lower frequency values, respectively, than the experimental ones, whereas the B3LYP-D3 data are systematically blue-shifted but for one mode (vide infra). A satisfactory agreement is also found when considering the previous FTIR results of Weir and Lippincott^30^ and the very recent ones of Bishop and collaborators^31^. The present simulations are also in good agreement with those of Ungureanu and co-workers^27^, who employed the same DFT functional (B3LYP) and basis sets but without any correction for long-range interactions. This could be the source of the observed differences, as in the case of the simulations performed by Carteret and collaborators^25^.

**Table 1:** Infrared-active vibrational frequencies (ν, in cm^−1^) of aragonite, subdivided into irreducible representations (IRREP).

IRREP	Mode		Theoretical				Experimental		
			B3LYP-D3^a^	B3LYP-D*^a^	B3LYP^b^	B3LYP^c^	^d^	^c^	^e^
*B*_1u_	L	1	197.0	180.4	174.6	174.1		183.1	
	L	2	232.7	213.6	202.5	210.1		207.8	
	L	3	276.7	267.2	270.1	269.1		259.2	
	L	4	309.9	291.0	291.9	288.9		286.9	
	ν_4_	5	726.8	719.3	714.3	719.2	715	718.3	717
	ν_2_	6	860.2	862.1	850.6	861.9	866	852.2	874
	ν_1_	7	1100.9	1091.6	1096.4	1092.9		1082.8	
	ν_3_	8	1491.0	1485.7	1488.6	1469.9			
*B*_2u_	L	1	90.0	27.8	75.1	65.4		105.4	
	L	2	175.9	163.0	158.9	158.7		164.2	
	L	3	229.6	202.0	197.2	198.0		219.9	
	ν_4_	4	703.5	697.7	692.2	697.4		699.8	
	ν_3_	5	1461.3	1443.4	1471.9	1445.1	1430	1444.5	1475
*B*_3u_	L	1	155.0	152.5	144.4	147.3		144.4	
	L	2	221.3	199.0	200.2	200.7		208.6	
	L	3	274.4	248.7	244.4	245.5		249.5	
	L	4	316.5	304.8	296.2	293.1		298	
	ν_4_	5	719.1	711.7	708.2	712.2	703	712.4	702
	ν_2_	6	917.6	915.2	914.7	913.1		908.8	
	ν_1_	7	1101.8	1092.5	1094.9	1092.9	1087	1082.8	1083
	ν_3_	8	1493.4	1486.7	1494.5	1474.1	1550	1466.6	1550
|Δ|max			26.8	20.1	27.9	21.9			
*MD*			13.4	2.8	1.5	0.2			
*MAD*			15.0	6.6	9.3	6.1			

Modes labelled as L are lattice modes, whereas ν_1_, ν_2_, ν_3_, and ν_4_ are CO_3_^2−^ vibrations.

^a^present work.

^b^DFT calculations performed by Ungureanu et al.^27^.

^c^DFT simulations and polarized IR spectroscopy data from the work of Carteret and co-workers^25^.

^d^Fourier infrared spectroscopy data of Weir and Lippincott^30^.

^e^Fourier infrared spectroscopy results from the work of Bishop and collaborators^31^.

However, the #1 and #3 modes of the *B*_2u_ IRREP calculated with the B3LYP-D* approach deviate from the experimental values^25^ by about − 78 cm^−1^ and − 18 cm^−1^ (red shifts), respectively. The same behaviour was noted for the same modes at the B3LYP level by both Ungureanu et al.^27^ and Carteret and co-workers^25^, who obtained red shifts of − 30 cm^−1^/− 23 cm^−1^ and − 40 cm^−1^/− 22 cm^−1^, respectively. It was suggested that these modes are very sensitive to the unit cell volume variations^25^, and the DFT-D3 results, for which the unit cell volume is smaller than both the B3LYP(-D*) and the experimental ones, seem to confirm this hypothesis because of the better agreement of the *B*_2*u*_ modes #1 and #3 with the polarized IR spectroscopy data (differences of − 15.4 cm^−1^ and 9.7 cm^−1^, respectively). This is an important issue, because these lattice modes, especially *B*_2u_ #1, could lead to phonon (dynamic) instability, as also previously explained by Ungureanu and collaborators^27^. However, the source of this discrepancy is probably elsewhere, as it will be discussed in the following.

In fact, linked to the IR vibrational data, the static dielectric tensor *ϵ*_0_ and its components (see Table 2) provided more insights into this matter. In general, the terms of the high frequency (electronic) dielectric tensor *ϵ*_∞_ are underestimated by about 3% (B3LYP-D3) and 5% (B3LYP-D*) with respect to the experimental values^25^, a common figure when employing hybrid functionals^7^. The static dielectric tensor is well described by the B3LYP-D3 approach, but strong variations were observed in the *xx* component of both *ϵ*_0_ and its ionic contribution *F* when using the B3LYP-D* method. This is due to the calculated oscillator strength of the #1 *B*_2u_ mode, which is very high compared to the experimentally determined value (see Table S2 in the Supplementary Materials). This overestimation, albeit of one order of magnitude lower than the present one at B3LYP-D*, was also obtained in the DFT/B3LYP simulations of Carteret and co-workers^25^. Hence, the trend in the DFT simulations, conducted with the same functional and localized basis sets, related to the oscillator strength of the #1 *B*_2u_ mode, was B3LYP-D3 (4.5254) < B3LYP (6.5549) < B3LYP-D* (54.048), with an experimental value of 2.2283.

**Table 2:** Static dielectric tensor *ϵ*_0_, as obtained from the sum of the high-frequency dielectric tensor *ϵ*_∞_ and the ionic contribution *F* (sum of the oscillator strengths *f*_*n*_). Δ% is the percentage difference between the B3LYP-D3 results and the experimental data.

	Method	*xx*	*yy*	*zz*
*ϵ*_0_	B3LYP-D3^a^	12.388	7.444	5.872
	B3LYP-D*^a^	62.909	8.258	6.370
	B3LYP^b^	15.588	8.087	6.406
	Experimental^b^	10.41	7.78	6.74
	Δ%	19.00	− 4.32	− 12.88
*ϵ*_∞_	B3LYP-D3^a^	2.740	2.753	2.243
	B3LYP-D*^a^	2.701	2.713	2.212
	B3LYP^b^	2.660	2.674	2.181
	Experimental^b^	2.81	2.82	2.33
	Δ%	− 2.48	− 2.39	− 3.75
*F* = Σ_*n*_*f*_*n*_	B3LYP-D3^a^	9.6478	4.6911	3.6294
	B3LYP-D*^a^	*60.21*	*5.545*	4.1576
	B3LYP^b^	*12.93*	*5.413*	4.225
	Experimental^b^	7.6	4.96	4.41
	Δ%	26.9	− 5.4	− 17.7

^a^present work.

^b^DFT/B3LYP results and experimental data from fitting of the reflectance spectrum from the work of Carteret and co-workers^25^.

**Figure 3:**
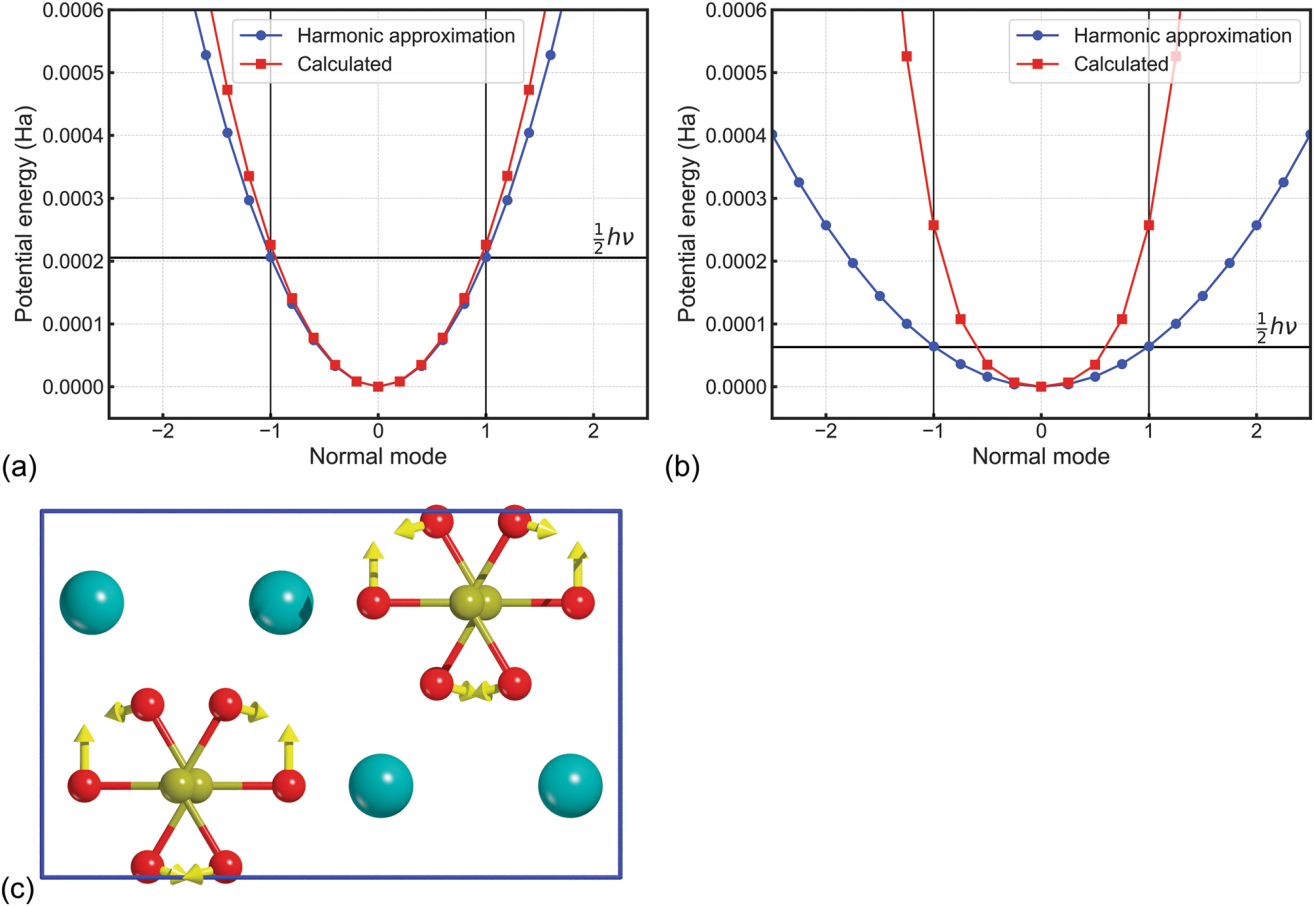
Potential energy well of the #1 *B*_2u_ optical mode (active in infrared) as obtained from (**a**) the B3LYP-D3 and (**b**) the B3LYP-D* approaches. The blue line corresponds to the harmonic approximation (see text for details), whereas the red line is the result of the scan performed along the displacement coordinate of the normal mode. The black thick horizontal line marks the fundamental vibrational energy level, calculated from the harmonic approximation (blue line). (**c**) Graphical representation of the eigenvectors (yellow arrows) of the #1 *B*_2u_ optical mode, with calcium, carbon and oxygen atoms in the unit cell coloured in blue, ochre and red, respectively.

To investigate these behaviours, it was performed a scan of the potential energy well on the cited *B*_2u_ optical mode, using both B3LYP-D3 and B3LYP-D* approaches (see Fig. 3). With this method, with **X**_0_ vector describing the equilibrium configuration of the system (Cartesian equilibrium coordinates of the atoms), we explored the configurations given by ${\mathbf{X}}_{n} = {\mathbf{X}}_{0} + n\Delta \times {\mathbf{L}}_{1}$, where **L**_1_ is the Cartesian eigenvector of the mode *B*_2u_ mode *Q*_1_, *n* is a positive or negative integer and Δ is the step along *Q*_1_. The maximum displacement corresponding to the classical turning points of the fundamental vibrational state is expressed by the equation:

$$V = \frac{1}{2}\omega_{1}^{2} Q_{1}^{2} = \frac{1}{2}k_{1} x_{1}^{2} = \frac{1}{2}\hbar \omega_{1} = E_{0}^{vib,1} ,$$

where *Q*_1_ is the displacement along the mass weighted *B*_2u_ normal mode coordinate #1, *ħ* is the reduced Planck’s constant, and $\nu_{1} = {{\omega_{1} } \mathord{\left/ {\vphantom {{\omega_{1} } {2\pi }}} \right. \kern-0pt} {2\pi }}$ is the vibrational frequency. In the above equation, $k_{1} = \mu_{1} \omega_{1}^{2}$ is the generalized force constant associated to the collective displacement coordinate $x_{1}$ (length units), $x_{1} = {{Q_{1} } \mathord{\left/ {\vphantom {{Q_{1} } {\sqrt {\mu_{1} } }}} \right. \kern-0pt} {\sqrt {\mu_{1} } }}$ with $\mu_{1}$ the reduced mass of the *Q*_1_ oscillator. Then, the maximum classical displacement in the fundamental level is given by the formula:

$$\left| {x_{1} } \right| = \sqrt {\frac{\hbar }{{\mu_{1} \omega_{1} }}} .$$

In Fig. 3, the displacement is reported as dimensionless normal coordinate $t_{1} = \left( {{{\omega_{1} } \mathord{\left/ {\vphantom {{\omega_{1} } \hbar }} \right. \kern-0pt} \hbar }} \right)^{{{1 \mathord{\left/ {\vphantom {1 2}} \right. \kern-0pt} 2}}} Q_{1}$, as provided by the CRYSTAL code, so at ± 1 the potential energy is equal to the energy of the fundamental vibrational state. From the graphs, it can be noted that in both B3LYP-D*n* simulations the shape of the potential energy curve calculated by scanning the normal mode coordinate (blue line) is a perfect parabola. In the B3LYP-D3 scan (red line in Fig. 3a), the potential well between − 1 and + 1 almost matches that of the harmonic potential, with a slight deviation at the extremes of this range, hence the computational parameters provide an adequate description of the *B*_2u_ optical mode. Conversely, the potential curve obtained from the B3LYP-D* simulation (Fig. 3b) deviates from the harmonic behaviour even below the classical displacement ± 1. These observed deviations could be due to some errors in the calculation of the Hessian matrix (second derivatives of the energy with respect to the atomic displacements) with the B3LYP-D* method. A possible source of errors could be the aragonite structure not being sufficiently close to the true minimum of the potential energy, but this hypothesis can be discarded because we adopted very strict convergence criteria during geometry optimization of the mineral in our previous work^28^. A second possibility could be the presence of large numerical errors during the computation of the Hessian. We are currently investigating this possibility, which is however beyond the scope of the present work. As an approximate solution, from the analysis of the potential energy surface, the force constant *k* of the harmonic oscillator was recalculated using the new data (red curve), finding a value that is higher than the previous one by an order of magnitude. This led to a vibrational frequency for this the #1* B*_2u_ mode of 103.6 cm^−1^, which is in line with the experimental data (105.4 cm^−1^). Following the same procedure with the B3LYP-D3 approach, we obtained a frequency of 102.6 cm^−1^, which is close to the theoretical value of 90.2 cm^−1^ reported in Table 1.

Regarding the Raman frequencies (see Table 3), as observed for the IR data, the simulations at the DFT/B3LYP-D3 and B3LYP-D* levels were in good agreement with the confocal Raman spectroscopy analysis on powdered aragonite performed in the present work (see Fig. 2b). The agreement can also be extended to previous experimental^10,26,34^ and theoretical studies^26,27^.

**Table 3:** Raman-active vibrational frequencies (ν, in cm^−1^) of aragonite, subdivided in irreducible representations (IRREP).

IRREP	Mode	Theoretical				Experimental			
		B3LYP-D3^a^	B3LYP-D*^a^	B3LYP^b^	B3LYP^c^	^a^	^d^	^e^	^10^
*A*_g_	L	150.5	146.9	147	148.7		144.4	144.7	143.5
	L	175.1	164.9	164.2	161.9		165.7	166.1	
	L	210.1	197.7	193.3	195.8		199	199.2	
	L	228.7	207.6	202.3	205		219.4	219.9	215.9
	L	306.4	285.9	283.3	280.2		291	291.5	284.8
	ν_4_	710.6	703.8	700.1	704.2	704.7	705	705.0	708.7
	ν_2_	861.3	863.1	851.5	862.8		853.8	854.0	
	ν_1_	1104.1	1095.1	1094.3	1095.3	1086.3	1087.2	1087.2	1086.4
	ν_3_	1489.7	1485.9	1492.1	1473.9			1466.2	
*B*_1g_	L	125.2	104.3	98.7	101.3		126.6	126.2	
	L	186.2	163.9	169.2	167.6		181	181.0	
	L	198.2	184.1	178.6	177.8		193.4	193.7	191.4
	L	293.8	284.9	274.1	271.4		278.4	278.5	274.4
	ν_4_	707.6	703.1	696.4	701.2	699.2	700	700.1	705.1
	ν_3_	1426.7	1410.4	1440.6	1415				
*B*_2g_	L	198.8	185.7	172.6	182.5	182.1	183.9	184.6	181.6
	L	221.4	213.7	210.3	207.2	209.4	212.3	212.8	207.9
	L	265.1	252.4	248.9	249.2		252.1	252.5	251.9
	L	283.7	264.9	259.5	260.7		266.1	267.0	262.3
	L	300.7	286.7	281.5	278.7			284.1	
	ν_4_	723.3	715.5	710.9	714.6	719.3	716.7	716.9	718.7
	ν_2_	916.3	913.9	913.5	911.8		918.7	911.1	
	ν_1_	1099.3	1091.1	1093.3	1091.6				
	ν_3_	1611.1	1603.4	1608.7	1591.8	1575.1	1579.1	1579.0	
*B*_3g_	L	117.8	93.8	96.6	97.4	114.5	117.6	117.6	113.3
	L	161.9	155.1	155.7	152.1	153.8	156.2	156.2	152.3
	L	227.9	202.8	200.4	199	208		211.7	
	L	237.5	224.2	214	213.4		226.5	227.0	
	ν_4_	712.4	707.2	700.6	705.5	707.9		705.9	
	ν_3_	1478.2	1461.7	1489.5	1463.9	1464.4	1464.1	1464.1	

Modes labelled as L are lattice modes, whereas ν_1_, ν_2_, ν_3_, and ν_4_ are CO_3_^2–^ vibrations.

^a^present work.

^b^DFT simulations of Ungureanu et al.^27^.

^c^DFT simulation performed by De La Pierre^26^.

^d^data obtained at 80 K on a polycrystalline sample^26^.

^e^data obtained at 80 K on a single-crystal sample^26^.

The effect of the long-range corrections on the lattice and carbonate ion modes is similar to that discussed for the infrared signals. The mean deviation (MD) and mean absolute deviation (MAD) of the B3LYP-D* frequencies with respect to the Raman measurements at 80 K of De La Pierre and co-workers^26^ are low (MD = − 1.1 and MAD = 6.9), and even lower than those resulting from the ab initio simulations carried out by the same authors (MD = − 4.6 and MAD = 7.7). In general, all the lattice and carbonate optical vibrations are within ± 10 cm^−1^ from the corresponding experimental values but for four low-frequency lattice modes (#4 *A*_g_, #1 and #2 *B*_1g_, #1 *B*_3g_) and two CO_3_^2^ vibrations (#9 *A*_g_ and #9 *B*_2g_) that showed an absolute deviation greater than 10 cm^−1^. These figures increase when employing the B3LYP-D3 approach, with mean (absolute) deviation of 10.6 (10.7) cm^−1^ with respect to the experimental data^26^, and a maximum absolute difference of 32.1 cm^−1^. Furthermore, as observed in the calculated infrared spectrum, the optical frequencies are systematically blue-shifted because of (i) the harmonic description of the modes and (ii) the smaller unit cell volume of aragonite, hence the increased interactions between the atoms in the structure. Indeed, previous X-ray diffraction determinations showed the aragonite cell volume being about 227 Å^3^ in room conditions (298 K and 1 atm)^19,35^, while DFT simulations without any thermal effect resulted in equilibrium geometries with cell volumes of ca. 236 Å^3^ (B3LYP)^25^, 230 Å^3^ (B3LYP-D*)^28^, and 223 Å^3^ (B3LYP-D3)^28^. As also explained in our previous works^7,28^, the inclusion of the dispersive forces significantly ameliorates the description of the geometry of both calcite and aragonite, resulting in unit cells that are slightly smaller than the experimental data.

Regarding the calculated intensities, it is possible to observe that the infrared signals are generally more intense on the ν_3_ carbonate ion modes (asymmetric stretching) whereas they are less strong in the spectral range 600–1100 cm^−1^. In the Raman spectra, the B3LYP-D*n* approach tends to overestimate the peak intensity of the ν_3_ in-plane O–C–O bending modes, and to underestimate the lattice modes (ν < 300 cm^−1^). In this latter case, on the experimental side, the spectrum could be affected by uncertainties associated to the measurements, whereas the different approximations in the simulations (basis sets, Hamiltonian, DFT-D*n* corrections and computational parameters) could be the source of the small differences. In this sense, we agree with the considerations of De La Pierre and collaborators^26^, who pointed out the basis set is the most probable cause of this discrepancy. In fact, albeit the quality of the chosen basis sets is indeed high for solid state calculations, Raman intensities may require even richer basis sets. However, while in molecular simulations there are very rich basis sets, such as aug-cc-pVTZ, Gaussian-type orbitals with this kind of accuracy have not been developed yet for periodic structures.

As a final note on the vibrational properties of aragonite at equilibrium conditions, the calculated frequencies of the silent *A*_*u*_ modes (four lattice and two carbonate ion vibrations, see Table S3 in the Supplementary Materials) are in line with those previously obtained by other authors^25,27^, with the DFT-D*n* corrections providing blue-shifted modes. As observed for the #1 *B*_2*u*_ mode active in infrared, the #1 *A*_*u*_ lattice mode strongly varies between the different simulation approaches, with the same frequency trend B3LYP-D3 > B3LYP > B3LYP-D*.

### Effect of pressure on the infrared and Raman properties of aragonite

The variation of the peaks in the vibrational spectra of aragonite as a function of (hydrostatic) pressure was investigated by calculating the optical frequencies and their IR/Raman intensities in unit cells of the mineral with different volumes. Each unit cell volume corresponds to a pressure value that was determined in a previous study by means of equation of state fitting^28^. The interested reader may find more information on this topic and detailed structural data of aragonite simulated with both B3LYP-D3 and B3LYP-D* approaches at various compressed/expanded states in the cited work.

The optical frequencies and their related signal (IR/Raman) intensities were calculated for 13 unit cell volumes, three in expansion and ten in compression, between − 3 GPa and 25 GPa. For the sake of brevity, the complete results of this procedure carried out with the B3LYP-D3 and B3LYP-D* approaches are reported in Table S4 and Table S5, respectively, in the Supplementary Materials. However, due to the observed anomalous behaviour of some calculated modes (vide supra), the following discussion will mainly consider the B3LYP-D3 results, and some comments on the B3LYP-D* data will be presented when applicable.

**Figure 4:**
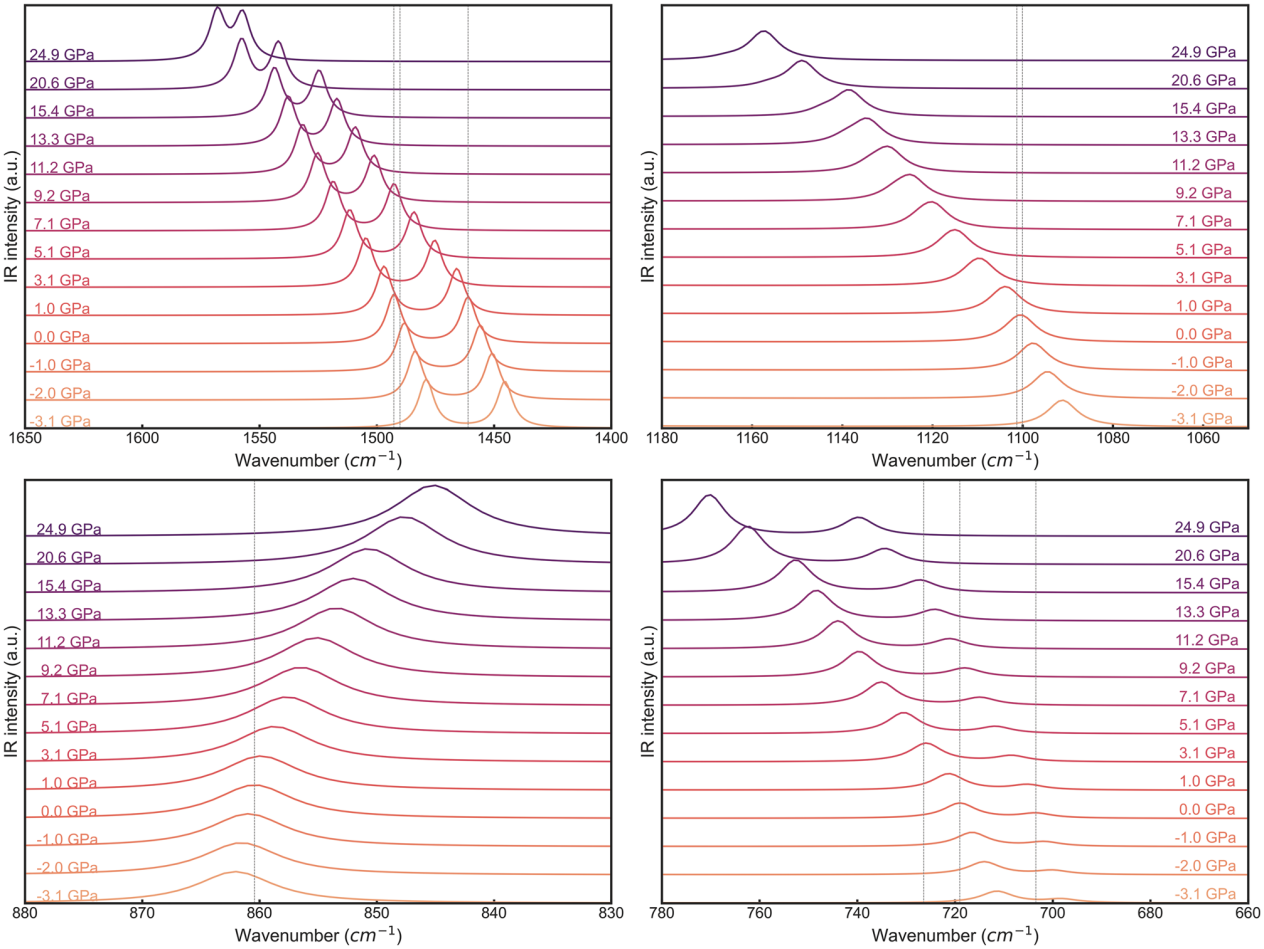
Evolution of the infrared spectrum of aragonite at different pressures, calculated at DFT/B3LYP-D3 level of theory. The spectra were subdivided in four regions, thus four panels, to avoid graphical cluttering and to better highlight the frequency shift of different IR bands. The vertical grey lines mark the position of the relevant peaks at 0 GPa. Infrared intensities are expressed in arbitrary units.

**Figure 5:**
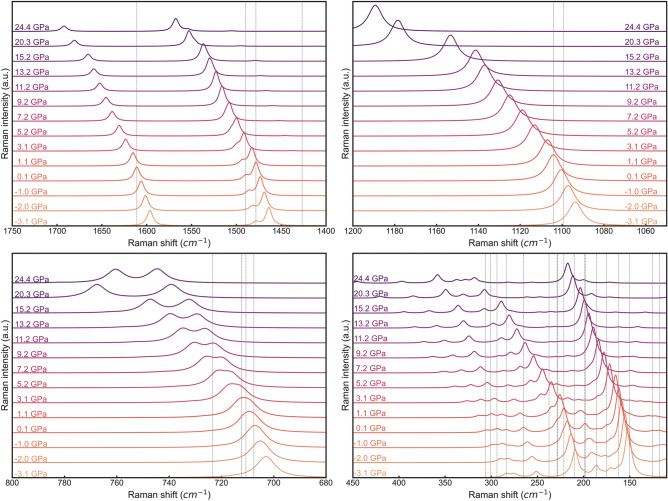
Evolution of the Raman spectrum of aragonite at different pressures, calculated at DFT/B3LYP-D3 level of theory. The spectra were subdivided in four regions, thus four panels, to avoid graphical cluttering and to better highlight the frequency shift of different IR bands. The vertical grey lines mark the position of the relevant peaks at 0 GPa. Raman intensities are expressed in arbitrary units.

A graphical representation of the evolution of the infrared and Raman spectra as a function of pressure is reported in Fig. 4 and Fig. 5, respectively, as obtained from the B3LYP functional corrected with the DFT-D3 scheme.

Qualitatively, it can be noted that the IR spectrum of aragonite, which is dominated by the CO_3_^2–^ modes in the range 2000–400 cm^−1^, is highly affected by pressure, with all but one optical mode (*ν*_2_ out-of-plane O–C–O bending mode with IRREP *B*_1u_) that are blue shifted by compression. The intensity of some signals is also enhanced due to (i) increased interatomic interactions, and hence dipole moment variation, (ii) overlapping of the peaks, or both. Similar considerations can be extended to the calculated Raman spectra where the symmetric stretching mode (*ν*_1_) of the CO_3_^2−^ ions and lattice modes with *ν* < 400 cm^−1^ are the most intense signals. The former peak shows a remarkable blue shift for *P* > 15 GPa, but no significant change in its intensity was observed. The single signal of the in-plane O–C–O bending mode (*ν*_4_) that falls at about 710 cm^−1^ is composed by two strong (*A*_g_ + *B*_1g_) and two very weak (*B*_2g_ + *B*_3g_) peaks that overlap. By increasing pressure, the two intense modes are blue shifted but with different ratio (*A*_g_ > *B*_1g_), resulting in the deconvolution of the single peak in two signals. Interestingly, the #6 *A*_g_ optical mode frequency increases up to about 21 GPa, then it decreases by further compression of the unit cell. This mode is related to a *ν*_4_ CO_3_^2−^ band, i.e., to O–C–O bending mode, which is graphically reported in Fig.S2 (Supplementary Materials). It is known that aragonite undergoes a phase transition to the post-aragonite structure (space group *Pmmn*) between 30 and 40 GPa^36,37^, and probably the change in the trend of this vibrational mode with pressure (at about 25 GPa) is a first sign of structural variations that will occur by further compressing the aragonite unit cell. Indeed, as shown in Fig.S2 (Supplementary Materials), by increasing pressure from 0 GPa up to 25 GPa, the carbonate ions move along the [001] direction. As recently observed by DFT simulations^38^, this is one of the possible change of the internal geometry of aragonite that leads to the post-aragonite structure. The frequency lowering of the cited vibrational mode could be due to the increased interaction between Ca^2+^ and CO_3_^2−^ ions that are brought closer due to pressure, which decreased the force constant of the O–C–O angle. Similarly to the *ν*_4_ carbonate mode, the lattice vibrations show high variations in the peak positions, intensity and overlapping of the signals.

A fruitful discussion can be also made by quantifying the blue and red shifts of the optical modes as a function of compression/expansion. There are several ways to describe the behaviour of phonon modes with pressure *P* (or volume Ω), which is of utmost importance when dealing with thermodynamics and thermoelasticity of crystalline phases^6,39–43^. For example, a simple polynomial function, as proposed by Erba and co-workers^44^ and recently adopted for several phases^43,45,46^, is typically sufficient to model the *ν*_*p*_(Ω) trend. Here, this functional form was described in terms of the so-called mode-Grüneisen (or mode-gamma) parameters, *γ*_*p*_, which are calculated for each *p*th mode as^47^:

$$\gamma_{p} = - \frac{{\partial \ln \nu_{p} }}{\partial \ln \Omega } = - \frac{\Omega }{{\nu_{p} }}\frac{{\partial \nu_{p} }}{\partial \Omega }.$$

**Figure 6:**
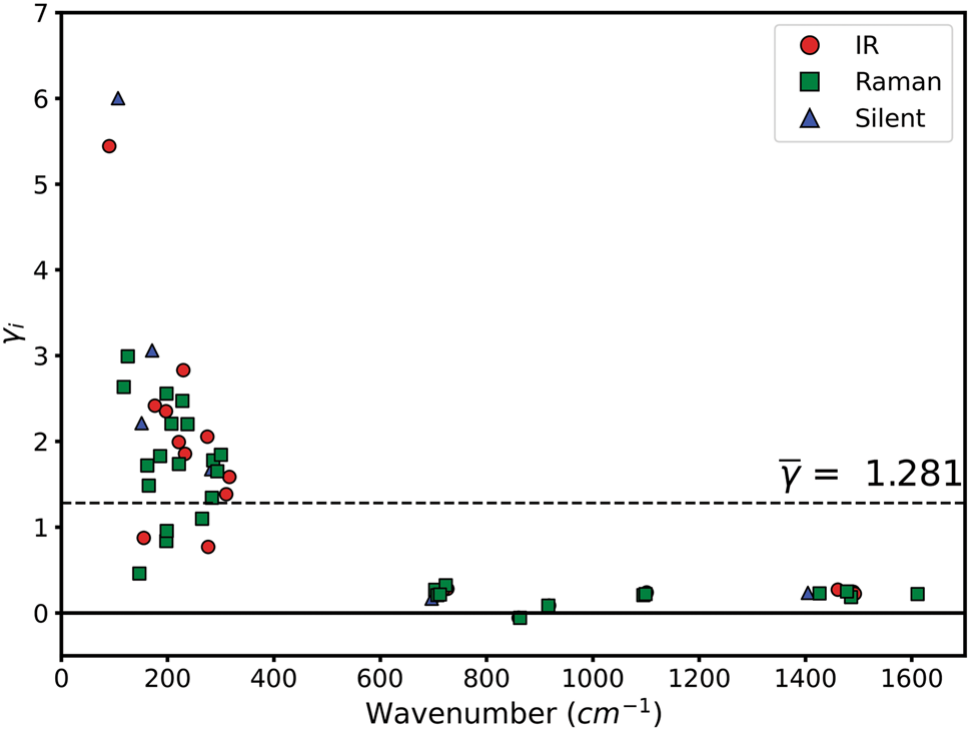
Mode-gamma Grüneisen’s parameters γ_*p*_ calculated at equilibrium volume at the B3LYP-D3 level, as a function of the vibrational frequency *ν*_*p*_.

For each normal mode, the *γ*_*p*_ parameter was calculated through the analytic first derivative at each volume (pressure) of the second order polynomial resulting from the fitting of the numerical *ν*_*p*_(Ω) curves. The resulting mode-Grüneisen parameters *γ*_*p*_ are reported in Table 4, and graphically shown in Fig. 6. It can be noted that most of the *γ*_*p*_ values are positive, meaning that the vibrational frequencies increase by reducing the unit cell volume, thus they increase with pressure. There are a few exceptions, *i.e.*, two *ν*_2_ CO_3_^2–^ optical modes (*B*_1u_ + *A*_g_) that showed the opposite behaviour. Lattice modes (L) have large mode-gamma parameters,* γ*_*p*_ > 1, whereas the carbonate ones have values close to zero (0–0.3), with a mean Grüneisen’s parameter $\overline{\gamma }$ equal to 1.281. These results are consistent with the crystal structure of aragonite, because the Ca–O interactions of the CaO_9_ polyhedrons showed a larger variation than the strong C–O bonds of the carbonate group with pressure^36,48,49^.

**Table 4:** Mode-Grüneisen parameters *γ*_*i*_ calculated at the equilibrium geometry for the infrared, Raman and silent optical frequencies of aragonite.

IRREP		B3LYP-D3^1^		B3LYP-D*^1^		B3LYP^2^		∆*γ*_*p*_	Experimental^3^	
		ν (cm^−1^)	*γ*_*p*_	ν (cm^−1^)	*γ*_*p*_	ν (cm^−1^)	*γ*_*p*_		ν (cm^−1^)	*γ*_*p*_
*B*_1u_	L	197.0	2.351	180.4	2.754	173.8	2.64	− 0.29		
	L	233.0	1.856	213.6	2.614	201.7	2.56	− 0.70		
	L	276.5	0.769	267.2	0.706	269.9	0.54	0.23		
	L	310.2	1.383	291.0	1.500	292.0	1.36	0.02		
	ν_4_	726.4	0.281	719.3	0.237	714.0	0.22	0.06		
	ν_2_	860.4	− 0.055	862.1	− 0.015	850.7	− 0.07	0.02		
	ν_1_	1100.0	0.220	1091.6	0.205	1092.1	0.16	0.06		
	ν_3_	1490.1	0.247	1485.7	0.273	1488.5	0.22	0.03		
*B*_2u_	L	90.2	5.442	27.8	28.375	72.7	9.86	− 4.42*		
	L	176.1	2.418	163.0	2.599	158.5	1.94	0.48		
	L	229.7	2.831	202.0	3.655	197.5	3.50	− 0.67		
	ν_4_	703.5	0.197	697.7	0.164	692.1	0.15	0.05		
	ν_3_	1461.0	0.271	1443.4	0.228	1468.5	0.20	0.07		
*B*_3u_	L	155.1	0.876	152.5	0.990	144.1	0.91	− 0.03		
	L	221.2	1.992	199.0	2.673	200.1	2.73	− 0.74		
	L	274.6	2.054	248.7	2.380	243.8	2.44	− 0.39		
	L	316.3	1.587	304.8	1.675	295.9	1.16	0.43		
	ν_4_	719.0	0.274	711.7	0.227	707.8	0.21	0.06		
	ν_2_	917.6	0.087	915.2	0.115	914.7	0.02	0.07		
	ν_1_	1101.3	0.238	1092.5	0.218	1091.9	0.18	0.06		
	ν_3_	1492.7	0.223	1486.7	0.226	1494.2	0.21	0.01		
*A*_g_	L	146.9	0.458	146.9	0.435	146.2	0.63	− 0.17		
	L	164.9	1.485	164.9	1.845	165.1	1.26	0.23		
	L	197.7	0.838	197.7	1.722	193.1	1.65	− 0.81		
	L	207.6	2.205	207.6	2.577	202.0	3.24	− 1.03		
	L	285.9	1.778	285.9	2.039	282.9	1.59	0.19		
	ν_4_	703.8	0.268	703.8	0.134	698.9	0.21	0.06		
	ν_2_	863.1	− 0.058	863.1	− 0.017	851.6	− 0.07	0.01		
	ν_1_	1095.1	0.207	1095.1	0.242	1094.0	0.19	0.02	1084	0.16
	ν_3_	1485.9	0.185	1485.9	0.200	1492.1	0.18	0.01		
*B*_1g_	L	125.2	2.991	104.3	4.766	99.3	5.72	− 2.73*		
	L	186.2	1.828	163.9	2.637	166.4	2.30	− 0.47		
	L	198.2	2.557	184.1	2.373	180.5	2.35	0.21	180	2.6
	L	293.8	1.652	284.9	1.928	274.2	1.33	0.32		
	ν_4_	707.6	0.206	703.1	0.183	696.1	0.15	0.06	702	0.14
	ν_3_	1426.7	0.227	1410.4	0.191	1437.2	0.17	0.06		
*B*_2g_	L	198.8	0.955	185.7	1.865	173.1	1.92	− 0.97		
	L	221.4	1.735	213.7	2.221	211.2	1.48	0.26	209	1.2
	L	265.1	1.097	252.4	1.107	249.2	1.42	− 0.32		
	L	283.7	1.342	264.9	1.465	258.3	1.48	− 0.14		
	L	300.7	1.842	286.7	1.801	281.5	1.26	0.58		
	ν_4_	723.3	0.321	715.5	0.272	710.3	0.25	0.07	710	0.21
	ν_2_	916.3	0.084	913.9	0.110	913.4	0.01	0.07		
	ν_1_	1099.3	0.218	1091.1	0.203	1090.5	0.17	0.05		
	ν_3_	1611.1	0.218	1603.4	0.209	1609.1	0.22	0.00		
*B*_3g_	L	117.8	2.635	93.8	4.327	95.4	5.28	− 2.64*		
	L	161.9	1.719	155.1	1.793	155.3	1.30	0.42	155	1.2
	L	227.9	2.471	202.8	3.315	201.6	2.77	− 0.30		
	L	237.5	2.200	224.2	2.139	214.1	2.07	0.13		
	ν_4_	712.4	0.213	707.2	0.190	700.6	0.18	0.03		
	ν_3_	1478.2	0.249	1461.7	0.202	1486.0	0.19	0.06		
*A*_u_	L	106.8	6.002	49.5	22.283	66.2	14.96	− 8.96*		
	L	151.2	2.212	138.5	1.765	132.9	3.08	− 0.87		
	L	170.9	3.060	150.2	3.969	146.9	3.41	− 0.35		
	L	281.9	1.676	273.5	1.862	265.6	1.14	0.54		
	ν_4_	696.9	0.166	691.2	0.121	686.8	0.13	0.04		
	ν_3_	1404.5	0.233	1388.1	0.198	1416.5	0.17	0.06		

^1^present work.

^2^DFT simulations of Ungureanu et al.^27^.

^3^Experimental Raman results of Gillet and collaborators^50^; ∆*γ*_*p*_ is the difference between the mode-gamma parameters calculated with B3LYP-D3 and B3LYP^27^ approaches. The asterisks mark ∆*γ*_*p*_ whose absolute values are greater than 1.5.

All these observations are in general good agreement with the previous theoretical analysis performed at the B3LYP level without the inclusion of van der Waals interactions^27^. In the present work, as expected, the use of a correction for the long-range interactions affected the trends of the variation of the optical mode frequencies with pressure, in particular for the lattice modes, with a mean absolute deviation from the values calculated by Ungureanu et al.^27^ of about 0.94. Conversely, the effect of the DFT-D3 scheme on the *γ*_*p*_ of the carbonate ion modes is much lower ($\left| {\overline{\Delta \gamma }_{p} } \right|$= 0.04). High differences in the mode-Grüneisen parameters were calculated for four lattice modes, marked with an asterisk in Table 4, which are associated to rotations of the carbonate ions around an axis parallel to the **c** lattice vector and centred on either the carbon or one of the oxygen atoms of the CO_3_^2−^ group. The highest deviations were observed for the #1 *B*_2u_, the one previously discussed, and the #1 *A*_u_, whose ∆*γ*_*p*_ values were − 4.42 and − 8.96, respectively. A graphical representation of the eigenvectors of the three vibrational modes not previously shown is reported in Fig.S1 in the Supplementary Materials.

The very large mode-gamma parameters previously calculated^27^ showed the occurrence of zone-central (Γ-point) soft phonons with negative frequency values, *i.e.*, the lattice became dynamically instable, when the aragonite unit cell is expanded, for example, by increasing temperature. According to their results, Ungureanu and collaborators^27^ suggested that there could be a phase transition at *P* = 0 GPa and *T* > 600 K. In the present study, the use of B3LYP-D3 approach did not provide any soft mode from –3 GPa to 25 GPa (see Table S4 in the Supplementary Materials), as the associated mode-Grüneisen parameters are small. Instead, our B3LYP-D* simulations reported in the present work provided *γ*_*p*_ for the #1 *B*_2u_ and the #1 *A*_u_ modes that are even larger than those obtained by Ungureanu and co-workers^27^, namely 28.38 (+ 18.52) and 22.28 (+ 7.32), respectively. This suggests that the aragonite-to-calcite phase transition would occur at lower temperatures than that previously hypothesized^27^. However, the considerations on any phase transition made upon the B3LYP-D* and B3LYP results have to be carefully addressed in future works.

Furthermore, the present mode-Grüneisen parameters at the B3LYP-D3 level well compare with the experimental ones of Gillet and collaborators^50^, obtained from Raman spectra collected at different pressure conditions (see Table 4). The same applies to the more recent results of Wang and co-workers^51^, who analysed by both FTIR and Raman spectroscopies the variation of the optical mode frequencies with pressure and temperature. The authors found that the mode-Grüneisen’s parameters are within 0.9–3.5 for lattice modes (*ν* < 350 cm^−1^) and below 0.4 for the carbonate ion vibrations (650–1600 cm^−1^), which are consistent with the present DFT simulations conducted with the inclusion of long-range interactions.

Aragonite (CaCO_3_, space group *Pmcn*) is one of the main polymorphs of calcium carbonate, with important applications in the fields of geology, mineralogy/petrology, biology and materials science. We can summarize the main findings of the present combined ab initio and experimental study on the vibrational (IR/Raman) properties of this mineral as follows:

- The inclusion of the long-range interactions via the DFT-D*n* schemes is relevant to the calculation of the vibrational properties of the mineral. The combination of the hybrid B3LYP functional and the DFT-D3 approach seems providing the best description of the physics of the system, providing accurate crystal-chemical, elastic, and vibrational spectroscopy data. The systematic blue shift, *i.e.*, increased frequency value, of the optical modes is a consequence of both the smaller unit cell volume calculated in static conditions (0 K without any thermal contribution) and the other necessary approximations in the simulations.
- The very high variability of the #1 *B*_2u_ (IR) and #1 *A*_u_ (silent) modes between B3LYP, B3LYP-D* and B3LYP-D3 calculations performed in the present work and in previous studies was analysed in detail. This variability is probably due to the computational parameters and methods employed for the calculation of the potential energy surface of the modes. In this context, it was found in the adopted approaches the trend B3LYP-D* < B3LYP < B3LYP-D3, with the latter providing better results in comparison with the experimental data, as also assessed by the analysis of the harmonic potential curve.
- The above consideration can be extended to other properties that depend on the lattice dynamics, such as the static dielectric tensor. In fact, the poorly defined oscillator strength of the *B*_2u_ mode resulted in a dramatic deviation of the *xx* component of the ionic and static dielectric tensors.
- The better vibrational results of the B3LYP-D3 approach ameliorated the description of the mode-Grüneisen’s parameters, in particular those of the cited *B*_2u_ and *A*_u_ modes, in better agreement with the few available experimental data.

## Methods

### Computational parameters

The ab initio simulations were carried out with the CRYSTAL17 software^32^, and within the Density Functional Theory framework. The Becke 3 parameters exchange functional^52^ and the correlation functional of Lee, Yang and Parr^53^ were adopted in the hybrid B3LYP formulation, which includes 20% of the exact Hartree–Fock (HF) exchange. In addition, B3LYP adds some non-local contribution to the exchange–correlation terms^32^. This hybrid functional is a common choice because of its high accuracy in determining the vibrational properties of both molecular and solid systems^33,54^. To properly consider the effects of long-range interactions, the semiempirical DFT-D2 scheme^55^, reparametrized according to the B3LYP-D* approach^56^, and the DFT-D3^57,58^ were employed throughout the work.

CRYSTAL17 employs a linear combination of atomic orbitals (LCAO) to construct the wave function of the system under analysis, and all-electron Gaussian-type orbitals (GTO) were selected for each atom in the aragonite structure among those optimized by Valenzano and co-workers^59^, named BSD in the cited work, which were adopted in the simulations of both aragonite^25,27^ and calcite^7–9^. More into details, 8–6511 (21), 6–311 (11) and 8–411 (11) basis sets were used for the description of the atomic orbitals of Ca, C and O, respectively.

The accuracy of Coulomb and exchange series are controlled by five thresholds, which were set to 10^–8^ (ITOL1 to ITOL4) and 10^−16^ (ITOL5), meaning that when the overlap between two atomic orbitals is lower than 10^−ITOL^, the corresponding integral is either discarded or treated with less precision. The first Brillouin zone (i.e., the reciprocal space) is sampled by means of a 6 × 6 × 6 Monkhorst–Pack mesh, corresponding to 64 independent *k* points^60^. The CRYSTAL default pruned grid (75 radial, 974 angular points, XLGRID) was used to calculate the total energy of the system, via numerical integration of the electron density over the unit cell volume^32^. Strict criteria on the self-consistent-field (SCF) convergence on energy was considered for the calculation of the vibrational frequencies, setting the threshold to 10^−10^ Hartree.

### Aragonite structural model

The aragonite model, simulated with the two DFT-D*n* schemes and under the effect of hydrostatic compression/expansion has been recently investigated by the authors of the present work in a recent theoretical study, using the same computational framework here adopted^28^. For the sake of completeness, the simulated unit cell parameters in equilibrium (zero pressure, absolute zero temperature) were *a* = 4.9544 Å, *b* = 7.8836 Å and *c* = 5.7091 Å (unit cell volume Ω = 222.987 Å^3^) at B3LYP-D3 level, and *a* = 5.0177 Å, *b* = 7.9005 Å and *c* = 5.7901 Å (Ω = 229.535 Å^3^) at B3LYP-D*.

### Calculation of the vibrational spectra

Vibrational frequencies of aragonite were obtained at Γ point at different pressures by diagonalizing the mass-weighted Hessian matrix *W* (dynamical matrix), whose elements are the second derivatives of the lattice potential with respect to mass-weighted atomic displacements^33^:

$$W_{\alpha i,\beta j} \left( \Gamma \right) = \frac{{H_{\alpha i,\beta j} }}{{\sqrt {M_{\alpha } M_{\beta } } }},$$

with *H*_*αi,βj*_ the energy second derivative, *M*_*α*_ and* M*_*β*_ the atomic masses and the subscripts in latin (*i*, *j*) and in Greek letters (*α*, *β*) the atomic coordinates and the atoms, respectively. The strength of the harmonic oscillators, *f*_*p*_, were calculated for each mode using the mass-weighted effective Born charge vectors $\vec{Z}_{n}$^25,61^:

$$f_{p,ij} = \frac{1}{{4\pi \varepsilon_{0} }}\frac{4\pi }{\Omega }\frac{{\vec{Z}_{n,i} \vec{Z}_{n,j} }}{{\nu_{p}^{2} }},$$

$$\vec{Z}_{p,i} = \sum\limits_{\alpha ,j} {{\mathbf{t}}_{p,\alpha j} Z_{\alpha ,ij}^{*} \frac{1}{{\sqrt {M_{\alpha } } }}} .$$

In the above formulas, *ϵ*_0_ is the dielectric permittivity in vacuum, *p* refers to the *p*th normal mode, *i* and *j* indicate the Cartesian components, and $Z_{\alpha }^{*}$ is the Born effective charge tensor of atom *α*, calculated using the Berry phase approach^62,63^. The term **t**_*n,αj*_ represent an element of the eigenvector matrix *T* that transforms the directions from Cartesian to normal coordinate.

Comparisons with previous experimental data was quantified according to the statistical indices previously adopted for calcite^7^:

$$\left| \Delta \right|_{\max } = \max \left( {\left| {\nu_{p} - \nu_{p}^{\exp } } \right|} \right)$$

$$MD = \frac{1}{M}\sum\limits_{p = 1}^{M} {\nu_{p} - \nu_{p}^{\exp } }$$

$$MAD = \frac{1}{M}\sum\limits_{p = 1}^{M} {\left| {\nu_{p} - \nu_{p}^{\exp } } \right|}$$

The infrared intensities were calculated analytically in terms of absorbance, using the classical absorption formula as explained by Maschio and co-workers^64^:

$$A\left( \nu \right) = \frac{1}{3}\sum\limits_{ii = 1}^{3} {\frac{4\pi }{{\lambda \rho }}{\text{Im}} \left[ {n_{ii} \left( \nu \right)} \right]} ,$$

where the terms *A*(*ν*), *λ*, *ρ*, and *n* are the infrared absorption, the wavelength of the incident light, the mineral density and the complex refractive index, respectively. The polarization direction is expressed by the subscripts *ii*. The real and imaginary parts of the refractive index *n*_*ii*_ were obtained from the following relations:

$$\left\{ {{\text{Re}} \left[ {n_{ii} \left( \nu \right)} \right]} \right\}^{2} - \left\{ {{\text{Im}} \left[ {n_{ii} \left( \nu \right)} \right]} \right\}^{2} = {\text{Re}} \left[ {\varepsilon_{ii} \left( \nu \right)} \right]$$

$$2{\text{Re}} \left[ {n_{ii} \left( \nu \right)} \right] \cdot {\text{Im}} \left[ {n_{ii} \left( \nu \right)} \right] = {\text{Im}} \left[ {\varepsilon_{ii} \left( \nu \right)} \right]$$

The term *ϵ*_*ii*_(*ν*) is the complex dielectric tensor, which was computed for each inequivalent polarization direction according to a classical Drude-Lorentz model:

$$\epsilon_{ii} \,\left( \nu \right)\, = \,\epsilon_{\infty ,ii} \, + \,\sum\limits_{p} {\frac{{f_{p,ii} \nu_{p}^{2} }}{{\nu_{p}^{2} - \nu^{2} - i\nu d_{p} }}} .$$

with *ϵ*_∞_ the optical (high frequency) dielectric tensor, *ν*_p_ the transverse optical frequency, *f*_p_ the oscillator strength and *d*_p_ the damping factor of the *p*th vibrational mode. As suggested by Maschio and co-workers^64^, to obtain a band broadening of the peaks similar to those typically found in experimental samples, the damping factor (i.e., the full width at half maximum of each vibrational mode) was set to 8. 

The Raman intensities were instead calculated within the Placzek approximation^65^ assuming the mineral as a polycrystalline powder, by using the optical vibrational modes by means of a pseudo-Voigt functional form^66,67^:

$$A\left( \nu \right) = \eta L\left( \nu \right) + \left( {1 - \eta } \right)G\left( \nu \right),$$

where, in this case, *A*(*ν*) represents the Raman intensity and* L*(*ν*) and* G*(*ν*) are given by:

$$L\left( \nu \right) = \sum\limits_{p} {\frac{{I_{p} }}{\pi }\frac{{{{\varphi_{p} } \mathord{\left/ {\vphantom {{\varphi_{p} } 2}} \right. \kern-0pt} 2}}}{{\left( {\nu - \nu_{p} } \right)^{2} + \left( {{{\varphi_{p} } \mathord{\left/ {\vphantom {{\varphi_{p} } 2}} \right. \kern-0pt} 2}} \right)^{2} }}}$$

$$G\left( \nu \right) = \sum\limits_{p} {2\sqrt {\frac{\ln 2}{\pi }} \frac{{I_{p} }}{{\varphi_{p} }}\exp \left[ { - \frac{{4\ln 2\left( {\nu - \nu_{p} } \right)^{2} }}{{\varphi_{p}^{2} }}} \right]}$$

with *I*_*p*_ the computed Raman intensity and *φ*_*p*_ the full width at half maximum for the *p*th vibrational mode, and *η* the Lorentz factor. A pure Lorentzian form, corresponding to *η* = 1, was employed to obtain the typical sharp peaks of Raman spectra^32^.

All the tensorial properties related to the intensity of the peaks in the infrared and Raman spectra, *i.e.*, the dielectric tensor, and the polarizability, were calculated analytically through a Couple Perturbed Kohn–Sham (CPKS) approach^68,69^. Finally, the static dielectric tensor was calculated as the sum of the high-frequency dielectric tensor *ϵ*_*∞*_ and the vibrational contribution, the latter expressed as the sum of all the oscillator strengths, $F_{{ij}} = \sum {_{n} f_{{n,ij}} }$^25^.

### Aragonite sample and Raman spectroscopy analyses

The aragonite CaCO_3_ sample used for the present study was a single-crystal specimen provided by the Mineralogical Museum “Bombicci” of the University of Bologna, Italy. The sample was grounded to a fine powder, with crystallite sizes below 1 μm, for the characterization of the vibrational properties via Raman spectroscopy. The Raman spectra were collected at room temperature (~ 20 °C) using a WITec alpha300R confocal Raman microscopy system, made of an optical microscope and an ultra-high throughput UHTS 300 VIS spectrometer with CCD camera and gratings of 600 g/mm. A green laser beam (532 nm) was used as excitation light, setting a power of 30 mW to prevent heating the sample and possible alterations on the aragonite phase (e.g., inducing phase transitions and decarbonisation of the sample). The laser beam was focused on the sample with a 20× Zeiss microscope objective with a low numerical aperture objective (NA = 0.40) to avoid optical artefacts. The backscattered Raman spectra were collected in confocal mode between 100 and 1700 cm^−1^, with a resolution of about 2.7 cm^−1^ and an acquisition time of 10 min. The Rayleigh scattering line was removed by an edge filter.

## Supplementary Information

Supplementary Information.

## Data Availability

The results of the present work are reported in the manuscript and in Supplementary Materials files. Relevant data that support the findings of this study are available from the corresponding author upon reasonable request.

## Code availability

For the present work, we employed the CRYSTAL17 software^32^ to perform the ab initio simulations, and the Raman spectra were processed with the WITec Project Five tools. Both CRYSTAL17 and WITec Project Five are commercially available programs.

## References

[1] Kristyán S, Pulay P (1994). Can (semi)local density functional theory account for the London dispersion forces?. Chem. Phys. Lett..

[2] Grimme S, Huenerbein R, Ehrlich S (2011). On the importance of the dispersion energy for the thermodynamic stability of molecules. ChemPhysChem.

[3] Cutini M, Corno M, Costa D, Ugliengo P (2019). How does collagen adsorb on hydroxyapatite? Insights from Ab initio simulations on a Polyproline type II model. J. Phys. Chem. C.

[4] Peccati F, Bernocco C, Ugliengo P, Corno M (2018). Properties and reactivity toward water of a type carbonated apatite and hydroxyapatite surfaces. J. Phys. Chem. C.

[5] Ulian G, Valdrè G (2018). Second-order elastic constants of hexagonal hydroxylapatite (P6_3_) from ab initio quantum mechanics: Comparison between DFT functionals and basis sets. Int. J. Quantum Chem..

[6] Ulian G, Valdrè G (2018). Equation of state of hexagonal hydroxylapatite (P6(3)) as obtained from density functional theory simulations. Int. J. Quantum Chem..

[7] Ulian G, Moro D, Valdrè G (2021). Benchmarking dispersion-corrected DFT methods for the evaluation of materials with anisotropic properties: Structural, electronic, dielectric, optical and vibrational analysis of calcite (CaCO_3_, space group: R-3c). Phys. Chem. Chem. Phys..

[8] Ulian G, Moro D, Valdrè G (2021). Elastic properties of heterodesmic composite structures: The case of calcite CaCO3 (space group R3¯c). Compos. C.

[9] Ulian G, Valdrè G (2022). Study of the variation of the optical properties of calcite with applied stress, useful for specific rock and material mechanics. Sci. Rep..

[10] Bayarjargal L, Fruhner CJ, Schrodt N, Winkler B (2018). CaCO_3_ phase diagram studied with Raman spectroscopy at pressures up to 50 GPa and high temperatures and DFT modeling. Phys. Earth Planet. Inter..

[11] Dasgupta R, Hirschmann MM (2010). The deep carbon cycle and melting in Earth’s interior. Earth Planet. Sci. Lett..

[12] Keppler H, Wiedenbeck M, Shcheka SS (2003). Carbon solubility in olivine and the mode of carbon storage in the Earth’s mantle. Nature.

[13] Deer WA, Howie RA, Zussman J (1992). An Introduction to the Rock-Forming Minerals.

[14] Farfan GA (2022). Crystallographic and chemical signatures in coral skeletal aragonite. Coral Reefs.

[15] Meldrum FC (2003). Calcium carbonate in biomineralisation and biomimetic chemistry. Int. Mater. Rev..

[16] Speight JG (2013). The Chemistry and Technology of Coal.

[17] Antao SM (2012). The crystal structure of a biogenic aragonite from the nacre of an ammonite shell. RSC Adv..

[18] Bragg WL (1924). The structure of aragonite. Proc. R. Soc. A.

[19] Caspi EN, Pokroy B, Lee PL, Quintana JP, Zolotoyabko E (2005). On the structure of aragonite. Acta Crystallogr. Sect. B.

[20] Muñoz-Moya E, García-Herrera CM, Lagos NA, Abarca-Ortega AF, Checa AG, Harper EM (2022). Evaluation of remodeling and geometry on the biomechanical properties of nacreous bivalve shells. Sci. Rep..

[21] Sibony-Nevo O (2022). The shell microstructure of the pteropod Creseis acicula is composed of nested arrays of S-shaped aragonite fibers: A unique biological material. MRS Bull..

[22] Adams A, Diamond LW (2017). Early diagenesis driven by widespread meteoric infiltration of a Central European carbonate ramp: A reinterpretation of the Upper Muschelkalk. Sediment. Geol..

[23] Gomez-Villalba LS, Feijoo J, Rabanal ME, Fort R (2021). In-situ electrochemical synthesis of inorganic compounds for materials conservation: Assessment of their effects on the porous structure. Ceram. Int..

[24] Toffolo MB, Ricci G, Caneve L, Kaplan-Ashiri I (2019). Luminescence reveals variations in local structural order of calcium carbonate polymorphs formed by different mechanisms. Sci. Rep..

[25] Carteret C, De la Pierre M, Dossot M, Pascale F, Erba A, Dovesi R (2013). The vibrational spectrum of CaCO_3_ aragonite: A combined experimental and quantum-mechanical investigation. J. Chem. Phys..

[26] De La Pierre M, Carteret C, Maschio L, André E, Orlando R, Dovesi R (2014). The Raman spectrum of CaCO3 polymorphs calcite and aragonite: A combined experimental and computational study. J. Chem. Phys..

[27] Ungureanu CG, Prencipe M, Cossio R (2010). Ab initio quantum-mechanical calculation of CaCO_3_ aragonite at high pressure: Thermodynamic properties and comparison with experimental data. Eur. J. Miner..

[28] Ulian G, Valdrè G (2022). Structural and elastic behaviour of calcium carbonate (CaCO_3_, space group *Pmcn*, aragonite) at high-pressure: A contribution from first-principle simulations. Comput. Mater. Sci..

[29] White WB, Farmer VC (1974). The carbonate minerals. The Infrared Spectra of Minerals.

[30] Weir CE, Lippincott ER (1961). Infrared studies of aragonite, calcite, and vaterite type structures in the borates, carbonates and nitrates. J. Res. Nat. Bur. Stand. Sect. A.

[31] Bishop JL (2021). Spectral properties of anhydrous carbonates and nitrates. Earth Space Sci..

[32] Dovesi R (2018). Quantum-mechanical condensed matter simulations with CRYSTAL. Wires Comput. Mol. Sci..

[33] Pascale F, Zicovich-Wilson CM, Gejo FL, Civalleri B, Orlando R, Dovesi R (2004). The calculation of the vibrational frequencies of crystalline compounds and its implementation in the CRYSTAL code. J. Comput. Chem..

[34] Alía JM, De Mera YD, Edwards HGM, González Martín P, López AS (1997). FT-Raman and infrared spectroscopic study of aragonite—Strontianite (CaxSr1—XCO3) solid solution. Spectrochim. Acta A.

[35] Antao SM, Hassan I (2009). The orthorhombic structure of CaCO_3_, SrCO_3_, PbCO_3_ and BaCO_3_: Linear structural trends. Can. Mineral..

[36] Ono S, Kikegawa T, Ohishi Y (2007). High-pressure transition of CaCO3. Am. Miner..

[37] Ono S, Kikegawa T, Ohishi Y, Tsuchiya J (2005). Post-aragonite phase transformation in CaCO3 at 40 GPa. Am. Miner..

[38] Salvadó MA, Pertierra P, Recio JM (2022). A mechanism for aragonite to post-aragonite transition in MCO_3_ (M = Ca, Sr and Ba) carbonates: Evidence of a hidden metastable polymorph. Phys. Chem. Chem. Phys..

[39] Belmonte D (2017). First principles thermodynamics of minerals at HP-HT conditions: MgO as a Prototypical material. Minerals.

[40] Ottonello G, Zuccolini MV, Civalleri B (2009). Thermo-chemical and thermo-physical properties of stishovite: An ab-initio all-electron investigation. CALPHAD.

[41] Prencipe M (2011). High-pressure thermo-elastic properties of beryl (Al_4_Be_6_Si_12_O_36_) from ab initio calculations, and observations about the source of thermal expansion. Phys. Chem. Miner..

[42] Ulian G, Valdrè G (2015). Density functional investigation of the thermophysical and thermochemical properties of talc Mg_3_Si_4_O_10_(OH)(2). Phys. Chem. Miner..

[43] Ulian G, Valdrè G (2019). Thermomechanical, electronic and thermodynamic properties of ZnS cubic polymorphs: An *ab initio* investigation on the zinc-blende – rock-salt phase transition. Acta Crystallogr. B.

[44] Erba A, Maul J, Demichelis R, Dovesi R (2015). Assessing thermochemical properties of materials through ab initio quantum-mechanical methods: The case of alpha-Al2O3. Phys. Chem. Chem. Phys..

[45] Ulian G, Moro D, Valdrè G (2021). Thermodynamic, elastic, and vibrational (IR/Raman) behavior of mixed type-AB carbonated hydroxylapatite by density functional theory. Am. Miner..

[46] Ulian G, Valdrè G (2020). First principle investigation of the thermomechanical properties of type A carbonated apatite. Int. J. Quantum Chem..

[47] Anderson OL (1995). Equation of State of Solids for Geophysics and Ceramic Science.

[48] Martinez I, Zhang J, Reeder RJ (1996). In situ X-ray diffraction of aragonite and dolomite at high pressure and high temperature: Evidence for dolomite breakdown to aragonite and magnesite. Am. Miner..

[49] Palaich SEM (2016). High-pressure compressibility and thermal expansion of aragonite. Am. Miner..

[50] Gillet P, Biellmann C, Reynard B, McMillan P (1993). Raman spectroscopic studies of carbonates part I: High-pressure and high-temperature behaviour of calcite, magnesite, dolomite and aragonite. Phys. Chem. Miner..

[51] Wang X (2019). High-temperature Raman and FTIR study of aragonite-group carbonates. Phys. Chem. Miner..

[52] Becke AD (1993). A new mixing of Hartree-Fock and local density-functional theories. J. Chem. Phys..

[53] Lee CT, Yang WT, Parr RG (1988). Development of the Colle-Salvetti correlation-energy formula into a functional of the electron-density. Phys. Rev. B.

[54] Pascale F, Zicovich-Wilson CM, Orlando R, Roetti C, Ugliengo P, Dovesi R (2005). Vibration frequencies of Mg_3_Al_2_Si_3_O_12_ pyrope. An ab initio study with the CRYSTAL code. J. Phys. Chem. B.

[55] Grimme S (2006). Semiempirical GGA-type density functional constructed with a long-range dispersion correction. J. Comput. Chem..

[56] Civalleri B, Zicovich-Wilson CM, Valenzano L, Ugliengo P (2008). B3LYP augmented with an empirical dispersion term (B3LYP-D*) as applied to molecular crystals. CrystEngComm.

[57] Grimme S, Antony J, Ehrlich S, Krieg H (2010). A consistent and accurate ab initio parametrization of density functional dispersion correction (DFT-D) for the 94 elements H-Pu. J. Chem. Phys..

[58] Grimme S, Ehrlich S, Goerigk L (2011). Effect of the damping function in dispersion corrected density functional theory. J. Comput. Chem..

[59] Valenzano L, Torres FJ, Klaus D, Pascale F, Zicovich-Wilson CM, Dovesi R (2006). Ab initio study of the vibrational spectrum and related properties of crystalline compounds; the case of CaCO_3_ calcite. Z. Phys. Chem..

[60] Monkhorst HJ, Pack JD (1976). Special points for Brillouin-zone integrations. Phys. Rev. B.

[61] Hess BA, Schaad LJ, ČÁRsky P, ZahradníK R (1986). Ab initio calculations of vibrational spectra and their use in the identification of unusual molecules. Chem. Rev..

[62] Baranek P, Zicovich-Wilson CM, Roetti C, Orlando R, Dovesi R (2001). Well localized crystalline orbitals obtained from bloch functions: The case of KNbO3. Phys. Rev. B.

[63] Noel Y, Zicovich-Wilson CM, Civalleri B, D’Arco P, Dovesi R (2002). Polarization properties of ZnO and BeO: An ab initio study through the Berry phase and Wannier functions approaches. Phys. Rev. B.

[64] Maschio L, Kirtman B, Orlando R, Rerat M (2012). Ab initio analytical infrared intensities for periodic systems through a coupled perturbed Hartree-Fock/Kohn-Sham method. J. Chem. Phys..

[65] Placzek G. *Handbuch der Radiologie*. Akademische Verlagsgeselschft (1934).

[66] Maschio L, Kirtman B, Rerat M, Orlando R, Dovesi R (2013). Ab initio analytical Raman intensities for periodic systems through a coupled perturbed Hartree-Fock/Kohn-Sham method in an atomic orbital basis. I. Theory. J. Chem. Phys..

[67] Maschio L, Kirtman B, Rerat M, Orlando R, Dovesi R (2013). Ab initio analytical Raman intensities for periodic systems through a coupled perturbed Hartree-Fock/Kohn-Sham method in an atomic orbital basis. II. Validation and comparison with experiments. J. Chem. Phys..

[68] Ferrero M, Rerat M, Kirtman B, Dovesi R (2008). Calculation of first and second static hyperpolarizabilities of one- to three-dimensional periodic compounds. Implementation in the CRYSTAL code. J. Chem. Phys..

[69] Ferrero M, Rerat M, Orlando R, Dovesi R (2008). The calculation of static polarizabilities of 1–3D periodic compounds. The implementation in the CRYSTAL code. J. Comput. Chem..

