# Supplementary material for: The effect of long-range interactions on the infrared and Raman spectra of aragonite (CaCO3, Pmcn) up to 25 GPa
Source: Sci Rep. 2023 Feb 15;13:2725. doi: 10.1038/s41598-023-29783-7 (PMC9932076; doi:10.1038/s41598-023-29783-7)
Supplement: Supplementary file 1 — Supplementary Information. [file 41598_2023_29783_MOESM1_ESM.pdf]

The effect of long-range interactions on the infrared and Raman spectra of aragonite ($CaCO_3$, *Pmcn*) up to 25 GPa

Gianfranco Ulian[1], Giovanni Valdrè[1,*]

1 Dipartimento di Scienze Biologiche, Geologiche e Ambientali, Centro di Ricerche Interdisciplinari di Biomineralogia, Cristallografia e Biomateriali, Università di Bologna "Alma Mater Studiorum" Piazza di Porta San Donato 1, 40126 Bologna, Italy.

Supplementary Materials

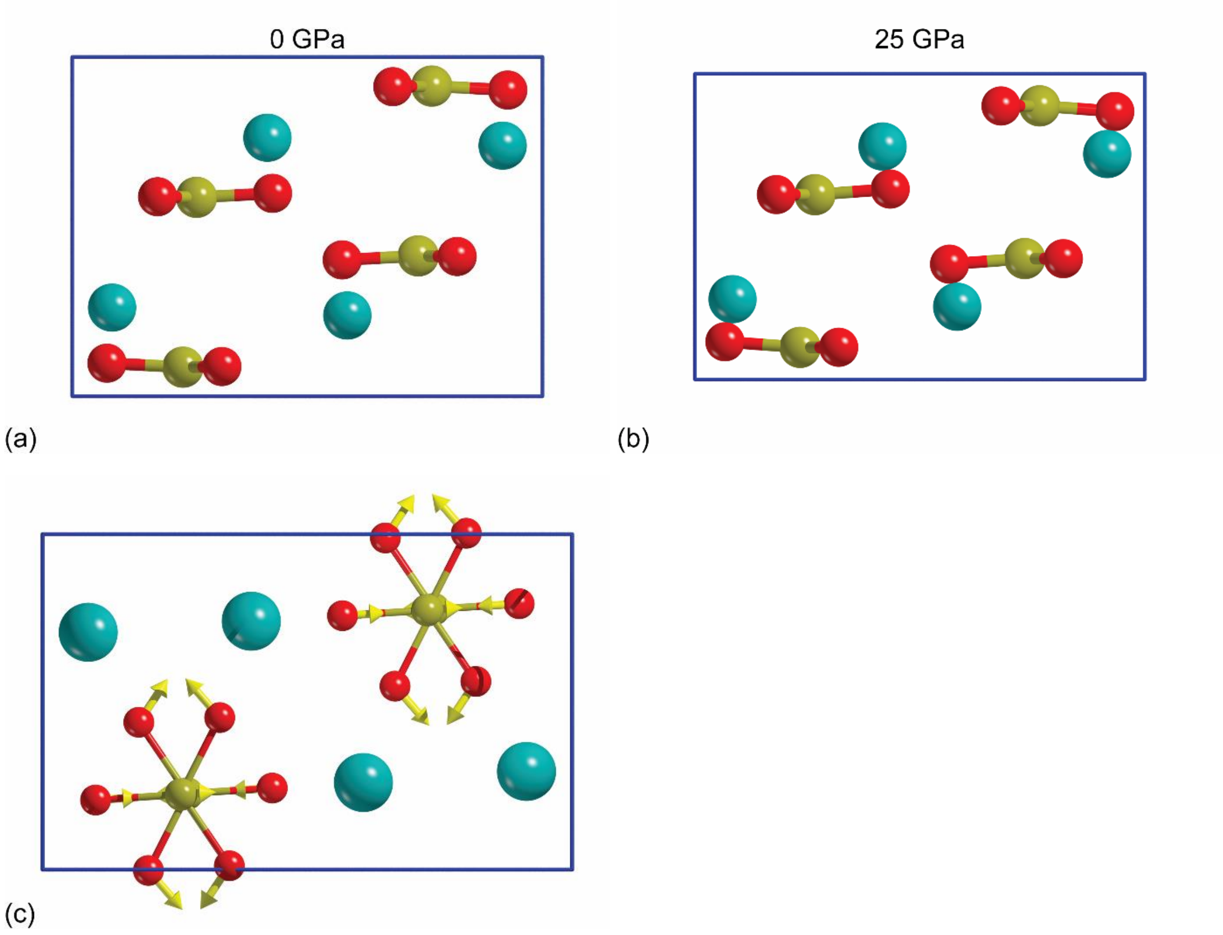

Figure S1. Views along the [100] direction of the configurations of aragonite (a) at 0 GPa and (b) at 25 GPa. It is worth noting the movements of the carbonate ions along the [001] direction (i.e., along the **c** axis). (c) The ν_4 CO_3^{2-} normal mode as viewed from the [001] direction (see text for details).

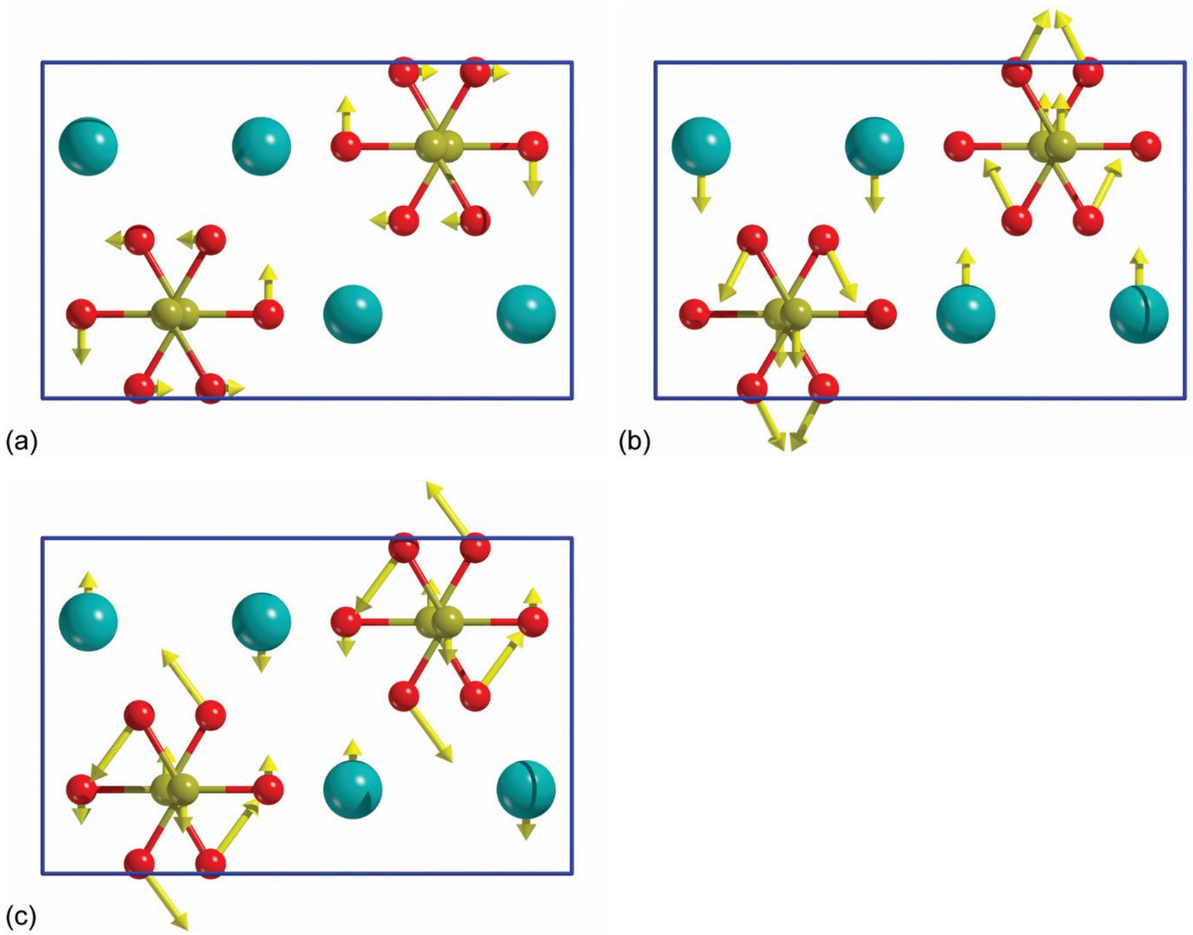

Figure S2. Selected normal modes showing very high variation in the mode-Grüneisen's parameters between B3LYP-D* and B3LYP-D3 methods. (a) A_u mode (silent), rotation of the carbonate ions around the C atom; (b) B_{3g} mode (Raman active), rotation of the carbonate ions around one of their O atoms; (c) B_{1g} mode (Raman active), rotation of the carbonate ions around one of their O atoms. In the three panels, Ca, C and O atoms are coloured in dark cyan, ochre and red, respectively, whereas the unit cell borders are represented with blue lines and the atom displacement vectors are shown as yellow arrows.

Table S1. Unit cell parameters (lattice constants and cell volume and mean relevant bond lengths and angles of aragonite as obtained from theoretical and experimental determinations.

	**B3LYP**[1]	**B3LYP-D***[2]	**B3LYP-D3**[2]	**XRD**[3]
a (Å)	5.008	5.0177	4.9544	4.96183
b (Å)	8.029	7.9005	7.8836	7.96914
c (Å)	5.861	5.7901	5.7091	5.74285
V ($Å^3$)	235.666	229.535	222.987	227.081
<C–O> (Å)	1.2847	1.2829	1.2857	1.289
<O–C–O> (°)	119.95	119.95	-	2.5272
<Ca --- O> (Å)	2.5402	2.5135	-	119.89

Table S2. Infrared vibrational modes ν (cm^{-1}) and oscillator strengths *f* (×10^3, dimensionless) from simulations and from experimental measurements and fittings.

		$\nu_{calc\text{-}D3}$[1]	$\nu_{calc\text{-}D*}$[2]	ν_{calc}[3]	ν_{exp}[3]	$\Delta\nu_{D3}$	$\Delta\nu_{D2}$	$f_{calc\text{-}D3}$[1]	$f_{calc\text{-}D*}$[2]	f_{calc}[3]	f_{exp}[3]	Δf_{D3}	Δf_{D*}
B_{1u}	L	197.0	180.4	174.1	183.1	13.9	-2.7	52.9	36.9	91.8	60.4	-7.5	-23.5
	L	232.7	213.6	210.1	207.8	24.9	5.8	3306.3	3795.6	3920.2	4105.6	-799.4	-310.0
	L	276.7	267.2	269.1	259.2	17.5	8.0	116.6	186.1	62.4	77.4	39.2	108.7
	L	309.9	291.0	288.9	286.9	23.0	4.1	21.7	18.8	35.0	43.5	-21.8	-24.7
	ν_4	726.8	719.3	719.2	718.3	8.5	1.0	0.3	0.2	0.1	0.4	-0.1	-0.2
	ν_2	860.2	862.1	861.9	852.2	8.0	9.9	128.7	117.2	113.1	116.1	12.6	1.1
	ν_1	1100.9	1091.6	1092.9	1082.8	18.1	8.8	2.9	2.7	2.4	1.9	1.0	0.8
	ν_3	1491.0	1485.7	1469.9	-	-	-	0.1	0.0	0.0	-	-	-
	$F[\Delta F]$							3629.4	4157.5	4225.0	4405.3	-775.9	-247.8
B_{2u}	L	90.0	27.8	65.4	105.4	-15.4	-77.6	4525.4	54048.2	6554.9	2228.3	2297.1	51819.9
	L	175.9	163.0	158.7	164.2	11.7	-1.2	1.2	1.0	62.1	53.4	-52.2	-52.4
	L	229.6	202.0	198	219.9	9.7	-17.9	4635.0	5675.7	5838.5	4862.0	-227.0	813.7
	ν_4	703.5	697.7	697.4	699.8	3.7	-2.1	6.7	4.1	5.2	6.7	0.0	-2.6
	ν_3	1461.3	1443.4	1445.1	1444.5	16.8	-1.1	479.5	478.7	467.1	458.0	21.5	20.7
	$F[\Delta F]$							9647.8	60207.7	12927.8	7608.4	2039.4	52599.3
B_{3u}	L	155.0	152.5	147.3	144.4	10.6	8.1	127.9	94.0	16.2	59.3	68.6	34.7
	L	221.3	199.0	200.7	208.6	12.7	-9.6	1911.1	2633.8	2617.5	1829.6	81.5	804.2
	L	274.4	248.7	245.5	249.5	24.9	-0.8	2147.7	2322.5	2286.7	2423.0	-275.3	-100.5
	L	316.5	304.8	293.1	298	18.5	6.8	0.0	0.1	7.0	164.9	-164.9	-164.8
	ν_4	719.1	711.7	712.2	712.4	6.7	-0.7	20.7	17.9	15.5	16.6	4.1	1.3
	ν_2	917.6	915.2	913.1	908.8	8.8	6.4	0.3	0.3	0.1	0.2	0.1	0.1
	ν_1	1101.8	1092.5	1092.9	1082.8	19.0	9.7	1.8	3.4	1.3	1.1	0.7	2.3
	ν_3	1493.4	1486.7	1474.1	1466.6	26.8	20.1	481.6	472.9	469.2	461.2	20.4	11.7
	$F[\Delta F]$							4691.1	5544.8	5413.5	4955.9	-264.8	588.9

Notes: 1 – present work, B3LYP-D3; 2 – present work, B3LYP-D*; 3 – B3LYP (calc) and experimental data from the work of Carteret et al.[1]. $\Delta\nu$ and Δf is the difference between the vibrational frequencies and oscillator strengths, respectively, simulated in the present work and the reported experimental ones. $F = \Sigma_n f_n$ is the sum of the oscillator strengths, whereas $\Delta F = \Sigma_n|\Delta f_n|$ is the sum of the absolute differences between the calculated and the experimental oscillator strengths.

Table S3. Silent vibrational modes ν (A_u, cm^{-1}) obtained from simulations at the DFT/B3LYP level of theory with and without corrections for the long-range interactions.

		**B3LYP-D3[1]**	**B3LYP-D*[1]**	**B3LYP[2]**	**B3LYP[3]**
A_u	L	106.8	49.5	70.8	69
	L	151.2	138.5	132.6	134
	L	170.9	150.2	147.6	143
	L	281.9	273.5	265.4	261
	ν_4	696.9	691.2	686.8	692
	ν_3	1404.5	1388.1	1420.1	1393

Notes: 1 – present work, 2 – theoretical study performed by Ungureanu et al.[4]; 3 – DFT simulations of Carteret and co-workers[1].

Table S4. Vibrational modes ν (cm^{-1}) of aragonite, subdivided in irreducible representations (IRREPs), simulated at the DFT/B3LYP-D3 level of theory between about –3 GPa and 25 GPa.

IRREP	Pressure (GPa)													
	-3.1	-2	-1	0	1	3.1	5.1	7.1	9.2	11.2	13.3	15.4	20.6	24.9
B_{1u}	177.8	184.6	190.9	197.0	202.7	213.5	224.1	233.9	243.6	252.6	261.3	269.8	289.2	302.6
	214.0	220.8	227.3	233.0	238.4	248.4	257.7	266.0	272.8	278.5	284.7	290.7	303.2	310.8
	269.2	271.6	274.0	276.5	279.1	284.5	289.9	295.5	300.8	306.0	311.2	316.6	330.4	340.8
	291.9	298.4	304.6	310.2	315.5	325.5	335.2	344.8	354.0	362.8	371.6	380.4	401.6	417.1
	718.7	721.4	724.1	726.4	729.1	733.6	738.6	743.0	747.4	752.0	756.3	760.5	769.8	777.5
	862.1	861.6	861.0	860.4	859.9	858.7	857.7	856.5	855.2	853.5	852.0	850.7	847.7	845.2
	1091.1	1094.3	1097.4	1100.0	1103.3	1109.1	1114.5	1119.7	1124.7	1129.7	1134.4	1138.4	1148.9	1157.4
	1475.3	1480.5	1485.6	1490.1	1494.8	1503.3	1510.7	1518.5	1525.2	1531.9	1538.0	1543.6	1555.9	1565.6
B_{2u}	62.8	73.7	82.7	90.2	96.6	107.9	116.9	124.4	130.8	137.0	142.0	146.5	154.9	160.1
	160.1	165.6	171.0	176.1	181.2	191.6	201.8	211.6	221.0	229.6	238.3	246.6	265.1	279.3
	201.9	211.7	221.0	229.7	237.7	253.1	267.3	280.4	292.3	303.4	313.8	323.5	344.3	360.3
	698.2	700.0	701.7	703.5	705.2	708.4	711.7	714.9	718.0	721.1	724.1	727.2	734.4	739.7
	1445.4	1450.8	1455.9	1461.0	1465.9	1475.3	1484.1	1492.6	1501.1	1509.1	1517.0	1524.6	1541.9	1557.2
B_{3u}	149.7	151.6	153.5	155.1	156.5	159.4	162.3	165.3	167.8	170.1	172.3	174.5	178.3	179.8
	202.3	209.3	215.5	221.2	226.5	236.3	246.1	255.4	263.6	270.9	278.2	285.7	301.5	310.3
	250.3	258.7	267.0	274.6	281.6	294.9	307.7	319.6	330.7	341.3	351.7	361.9	386.9	404.5
	297.0	303.6	310.0	316.3	322.4	334.7	346.3	357.4	367.9	378.0	387.7	397.3	419.1	435.9
	711.4	714.0	716.5	719.0	721.3	725.9	730.5	735.1	739.6	744.0	748.4	752.6	762.4	770.3
	914.7	915.8	916.6	917.6	918.6	920.6	922.6	924.4	926.1	927.5	929.1	930.6	934.3	938.0
	1091.4	1094.8	1098.3	1101.3	1104.8	1111.0	1116.6	1122.5	1128.2	1133.7	1139.1	1144.1	1156.1	1165.6
	1478.9	1483.6	1488.2	1492.7	1496.9	1504.6	1511.5	1518.6	1525.2	1531.6	1537.8	1543.6	1557.6	1568.0
A_g	149.8	150.4	150.4	150.5	150.2	149.6	148.5	148.0	146.3	143.3	141.4	133.2	107.5	82.6
	162.8	167.4	171.4	175.1	178.3	184.6	189.4	193.1	195.6	196.2	198.1	196.1	191.5	199.7
	199.4	204.0	207.4	210.1	212.6	216.6	220.8	225.7	230.2	235.2	239.6	248.1	261.4	281.9
	208.9	215.6	222.3	228.7	235.0	246.5	257.6	268.7	279.1	289.0	298.0	307.7	330.3	337.6
	283.8	291.3	299.1	306.4	313.2	326.9	340.1	352.1	363.5	373.2	383.0	394.6	420.6	445.1
	703.6	706.0	708.1	710.6	713.0	717.2	721.7	726.2	730.8	735.1	739.8	747.3	767.6	760.5
	863.0	862.4	861.8	861.3	860.8	859.6	858.5	857.3	856.0	853.1	853.3	853.1	849.6	847.3
	1093.7	1097.1	1100.4	1104.1	1107.0	1113.2	1118.9	1125.0	1130.7	1137.0	1141.3	1153.3	1178.5	1189.3
	1478.2	1482.2	1486.1	1489.7	1493.4	1499.7	1505.4	1511.5	1517.0	1522.0	1527.1	1533.8	1546.8	1554.4
B_{1g}	105.4	113.0	119.7	125.2	129.9	138.1	144.9	150.5	155.1	158.9	162.3	165.3	171.6	172.4
	170.9	177.2	182.0	186.2	190.2	197.8	205.3	212.3	218.6	224.7	230.4	235.7	247.5	256.1
	179.5	185.1	191.6	198.2	204.6	216.9	228.6	239.6	249.7	259.3	268.4	277.1	296.2	309.1
	275.2	281.5	287.8	293.8	299.6	311.2	322.2	332.6	342.0	351.0	359.4	367.3	384.6	396.9
	702.2	704.0	705.8	707.6	709.3	712.7	716.1	719.5	722.7	726.0	729.1	732.2	739.0	744.4
	1414.0	1418.4	1422.5	1426.7	1430.7	1438.4	1445.7	1452.7	1459.6	1466.4	1472.9	1478.9	1495.3	1505.4
B_{2g}	186.5	191.5	195.6	198.8	201.4	205.4	209.4	212.8	215.9	218.8	222.0	225.6	236.8	247.2
	209.2	213.2	217.3	221.4	225.8	234.9	244.5	254.3	263.4	272.3	280.6	289.2	307.4	318.1
	251.1	256.1	260.8	265.1	268.8	275.7	281.5	287.1	292.1	296.8	302.0	307.0	319.0	328.1
	265.3	272.2	278.4	283.7	288.5	296.7	304.5	311.8	318.4	324.2	330.1	336.0	349.3	358.3
	283.0	288.5	294.3	300.7	307.2	321.5	335.0	348.6	361.2	373.4	385.0	396.1	421.7	440.7
	714.4	717.5	720.5	723.3	726.1	731.5	737.0	742.2	747.5	752.5	757.5	762.3	773.8	782.4
	913.5	914.5	915.3	916.3	917.4	919.1	921.1	922.8	924.4	925.8	927.3	928.7	932.2	935.5
	1090.0	1093.2	1096.3	1099.3	1102.4	1108.0	1113.1	1118.3	1123.3	1128.1	1132.7	1136.9	1145.9	1154.7
	1596.4	1601.4	1606.2	1611.1	1615.3	1623.5	1630.7	1638.5	1645.4	1652.4	1659.0	1665.3	1680.7	1692.3
B_{3g}	101.1	107.8	113.3	117.8	121.7	128.3	134.0	139.3	144.0	148.4	152.3	156.0	165.6	168.5
	151.9	155.3	158.8	161.9	165.3	171.8	178.0	184.0	189.4	194.4	199.2	203.5	211.6	217.1
	204.3	212.7	220.6	227.9	234.7	247.7	260.0	271.5	281.9	291.7	301.0	309.6	325.7	340.0
	217.3	224.2	230.9	237.5	243.9	256.9	269.1	280.5	290.9	300.8	310.2	319.2	340.5	356.7
	706.6	708.6	710.5	712.4	714.2	717.7	721.1	724.4	727.5	730.4	733.3	736.1	742.3	746.5
	1463.8	1468.8	1473.4	1478.2	1482.7	1491.5	1499.7	1507.6	1515.8	1523.0	1530.3	1537.2	1552.9	1567.6
A_u	71.7	85.8	97.1	106.8	115.3	130.2	142.3	152.2	160.2	166.9	172.4	177.1	185.3	190.0
	136.3	141.5	146.6	151.2	155.4	163.5	171.1	178.7	186.1	193.8	201.3	209.0	227.5	242.9
	148.6	156.7	164.0	170.9	177.3	189.4	201.2	212.2	222.4	232.1	241.6	250.4	269.1	283.2
	264.4	270.3	276.1	281.9	287.5	299.0	309.9	320.3	330.0	339.3	347.9	356.3	374.8	387.9
	692.6	694.1	695.5	696.9	698.3	701.1	703.7	706.3	708.8	711.4	713.9	716.4	722.0	725.9
	1391.7	1396.2	1400.3	1404.5	1408.6	1416.4	1423.7	1430.6	1437.4	1444.5	1451.0	1457.2	1471.1	1483.9

Table S5. DFT/B3LYP-D* vibrational modes ν (cm^{-1}) of aragonite, subdivided in irreducible representations (IRREPs), between about –3 GPa and 25 GPa. Frequency marked with an asterisk are soft modes.

IRREP	Pressure (GPa)													
	-3.1	-2.0	-1.0	0.1	1.1	3.1	5.2	7.2	9.2	11.2	13.2	15.2	20.3	24.4
B_{1u}	155.2	164.6	172.9	180.4	187.4	200.1	211.9	222.7	232.3	241.1	249.4	257.1	274.8	289.1
	187.4	197.2	205.8	213.6	220.3	232.5	244.3	254.9	264.8	273.0	280.4	287.5	304.6	308.8
	259.2	261.8	264.4	267.2	269.7	275.2	280.9	287.0	292.8	297.8	302.9	307.1	319.6	330.0
	269.1	276.7	284.2	291.0	297.3	309.1	320.3	330.9	340.9	349.8	358.7	367.1	386.8	402.3
	710.8	713.6	716.5	719.3	721.8	726.9	731.9	736.6	741.1	745.3	750.2	754.0	764.1	772.1
	864.2	863.5	862.9	862.1	861.8	860.3	859.3	858.2	857.2	855.7	854.0	851.7	848.8	844.5
	1081.5	1085.1	1088.3	1091.6	1094.1	1100.6	1106.4	1111.9	1117.3	1122.2	1127.6	1131.6	1142.4	1150.5
	1467.4	1473.9	1479.9	1485.7	1491.0	1500.9	1510.3	1518.6	1526.7	1533.6	1540.3	1546.1	1560.7	1569.1
B_{2u}	-56.3*	-40.9*	-18.4*	27.8	44.0	63.2	75.6	87.1	96.2	104.0	111.5	117.7	130.2	140.9
	145.7	151.9	157.3	163.0	168.5	179.2	190.0	200.4	210.2	218.8	227.5	235.6	254.8	267.1
	167.4	180.0	191.5	202.0	212.1	230.1	246.8	261.7	275.5	288.2	300.1	310.6	335.6	350.9
	692.0	694.0	695.9	697.7	699.5	703.0	706.3	709.6	712.8	715.8	718.7	721.5	728.6	734.4
	1426.8	1432.5	1438.3	1443.4	1448.6	1458.2	1467.1	1476.4	1485.1	1493.1	1501.0	1509.0	1527.2	1542.5
B_{3u}	143.6	147.4	150.2	152.5	154.5	157.8	161.4	164.7	167.8	170.1	172.7	174.4	180.5	182.9
	173.4	182.5	191.2	199.0	205.7	217.9	229.9	241.4	251.9	260.8	269.0	276.8	295.6	304.9
	219.7	230.1	239.6	248.7	257.0	272.5	287.0	300.2	312.6	323.4	333.9	343.5	366.7	385.1
	283.8	290.8	297.9	304.8	311.4	324.6	337.5	349.5	360.8	371.4	381.5	391.1	413.0	428.2
	703.5	706.3	709.1	711.7	714.2	719.1	723.9	728.6	733.2	737.6	742.0	746.2	756.7	764.6
	911.7	912.8	914.3	915.2	916.7	918.5	920.8	922.8	924.8	926.4	927.4	928.5	931.0	932.5
	1081.7	1085.3	1089.0	1092.5	1095.6	1102.4	1108.8	1115.0	1120.8	1126.3	1131.7	1136.6	1148.8	1158.1
	1470.8	1476.4	1481.6	1486.7	1491.3	1500.1	1508.3	1515.9	1523.0	1529.4	1535.5	1541.0	1554.8	1564.6
A_{g}	139.6	144.0	146.2	146.9	147.0	146.7	146.5	146.4	145.8	144.6	142.6	140.8	138.6	130.3
	154.9	156.3	160.3	164.9	168.3	175.4	180.5	184.0	186.9	189.0	190.8	193.4	194.0	195.4
	174.1	183.8	191.5	197.7	202.1	210.3	218.1	224.9	231.6	237.1	241.2	245.3	258.5	263.9
	186.0	193.5	200.5	207.6	214.2	227.8	240.5	252.8	264.3	274.7	284.3	292.5	318.2	327.4
	261.3	269.8	278.2	285.9	293.6	308.1	322.7	336.3	349.1	360.5	372.1	382.0	405.5	423.2
	696.0	698.8	701.4	703.8	706.1	710.6	715.4	720.1	724.3	728.7	729.5	737.6	745.8	762.1
	865.2	864.5	863.9	863.1	862.8	861.4	860.5	859.3	858.3	856.9	855.2	849.3	850.1	844.7
	1084.1	1087.8	1091.6	1095.1	1098.9	1105.2	1111.9	1117.6	1123.4	1128.9	1133.1	1140.8	1151.5	1156.1
	1472.2	1477.0	1481.5	1485.9	1489.9	1497.6	1504.8	1511.4	1517.6	1523.0	1528.8	1532.8	1546.8	1552.8
B_{1g}	70.9	85.4	96.1	104.3	111.4	122.9	132.2	139.6	146.4	151.7	156.1	161.0	170.4	174.2
	136.1	146.6	155.9	163.9	171.2	183.1	191.6	198.9	205.3	211.3	217.1	222.2	234.1	243.1
	172.9	176.3	179.8	184.1	188.6	199.1	211.2	223.3	235.0	245.8	255.9	265.9	288.2	302.4
	262.5	270.2	277.6	284.9	291.7	305.0	317.8	329.4	340.4	350.1	359.5	367.9	387.2	399.2
	697.0	699.2	701.1	703.1	705.0	708.7	712.3	715.9	719.4	722.6	725.8	728.9	736.6	741.9
	1397.3	1402.1	1406.6	1410.4	1414.3	1422.6	1429.9	1437.6	1444.2	1451.4	1458.5	1464.6	1479.6	1492.7
B_{2g}	163.4	172.4	179.6	185.7	190.3	197.6	204.0	209.3	214.0	217.2	219.6	221.3	225.8	229.8
	199.0	203.7	208.5	213.7	218.9	229.5	240.9	251.3	261.2	269.4	277.8	285.4	302.9	310.8
	234.1	240.8	246.8	252.4	257.2	265.7	273.0	279.0	284.5	289.3	293.6	298.2	310.7	321.8
	245.5	252.5	258.9	264.9	270.3	280.2	289.5	298.4	306.9	314.0	321.1	327.6	344.0	354.2
	267.7	273.9	280.3	286.7	293.2	306.9	320.7	334.3	347.6	360.5	372.5	384.1	410.2	429.0
	705.4	709.0	712.3	715.5	718.5	724.2	730.0	735.4	740.7	745.8	751.0	755.8	767.7	776.8
	910.8	911.8	913.0	913.9	915.1	917.0	919.2	921.1	923.0	924.5	925.6	926.7	929.1	930.5
	1081.2	1084.5	1087.9	1091.1	1093.8	1100.0	1105.7	1111.2	1116.3	1121.3	1126.2	1130.3	1140.9	1148.6
	1586.8	1592.6	1598.2	1603.4	1608.3	1617.4	1625.9	1633.6	1641.0	1647.7	1654.1	1660.1	1674.8	1686.1
B_{3g}	62.6	75.8	85.9	93.8	100.1	110.0	118.3	125.5	132.1	138.1	143.5	149.2	160.1	166.8
	143.6	147.9	151.3	155.1	158.5	165.2	171.6	177.7	183.4	187.9	192.4	196.6	205.9	212.7
	173.5	184.0	193.6	202.8	211.5	227.6	242.5	256.1	268.3	279.3	289.4	298.9	319.9	333.0
	203.4	210.4	217.6	224.2	230.9	243.7	255.8	267.4	278.5	288.6	298.3	308.1	329.5	345.4
	701.0	703.2	705.2	707.2	709.1	712.8	716.4	719.8	723.2	726.3	729.3	732.2	738.9	744.1
	1446.5	1451.7	1457.0	1461.7	1466.5	1475.3	1483.1	1492.0	1500.0	1507.3	1514.9	1522.1	1538.9	1553.6
A_{u}	-63.8*	-41.6*	20.0	49.5	66.2	91.3	109.7	126.0	139.2	150.7	161.2	170.1	186.7	193.2
	115.2	125.8	132.9	138.5	143.5	151.8	159.2	165.7	171.6	176.8	181.9	187.8	202.3	217.1
	124.8	132.7	141.5	150.2	158.4	173.4	187.3	199.6	211.4	221.8	231.9	241.1	263.0	276.6
	253.8	260.3	266.8	273.5	279.8	292.3	304.9	316.3	327.2	337.0	346.6	355.5	375.4	388.4
	686.8	688.4	689.8	691.2	692.6	695.3	697.9	700.6	703.2	705.7	708.4	710.8	717.2	721.8
	1374.7	1379.5	1384.1	1388.1	1392.2	1400.5	1407.9	1415.8	1422.6	1429.9	1436.7	1443.3	1458.2	1471.6

References

1. Carteret C, De la Pierre M, Dossot M, Pascale F, Erba A, Dovesi R. The vibrational spectrum of $CaCO_3$ aragonite: A combined experimental and quantum-mechanical investigation. *Journal of Chemical Physics* **138**, 014201 (2013).
2. Ulian G, Valdrè G. Structural and elastic behaviour of calcium carbonate ($CaCO_3$, space group *Pmcn*, aragonite) at high-pressure: a contribution from first-principle simulations. *Computational Materials Science* **212**, 111600 (2022).
3. Caspi EN, Pokroy B, Lee PL, Quintana JP, Zolotoyabko E. On the structure of aragonite. *Acta Crystallogr Sect B Struct Sci* **61**, 129-132 (2005).
4. Ungureanu CG, Prencipe M, Cossio R. Ab initio quantum-mechanical calculation of $CaCO_3$ aragonite at high pressure: thermodynamic properties and comparison with experimental data. *Eur J Mineral* **22**, 693-701 (2010).
